# A Locally Injectable, pH/ROS-Responsive Hydrogel Platform for Combination Therapy of Cervical Cancer with Anti-Fibrotic and Chemotherapeutic Agents

**DOI:** 10.3390/pharmaceutics18091115

**Published:** 2026-09-04

**Authors:** Qian Chen, Hui Yang, Meili Pei, Yanxia Sun, Yubei Li, Sen Yu, Xiaofeng Yang

**Affiliations:** 1 Department of Gynecology and Obstetrics, The First Affiliated Hospital of Xi’an Jiaotong University, Xi’an 710061, China; qian@stu.xjtu.edu.cn (Q.C.); peiml@xjtufh.edu.cn (M.P.); sunyanxia@stu.xjtu.edu.cn (Y.S.); liyubei@stu.xjtu.edu.cn (Y.L.); 2 Shaanxi Key Laboratory of Biomedical Metallic Materials, Northwest Institute for Non-Ferrous Metal Research, Xi’an 710014, China; huiyang@c-nin.com

**Keywords:** cervical cancer, dual-responsive hydrogel, cancer-associated fibroblasts, tumor microenvironment, homotypic targeting nanoparticles

## Abstract

**Background:** The fibrous tumor extracellular matrix (ECM), driven by cancer-associated fibroblasts (CAFs), forms a physical barrier against drugs and immune cells, yet direct CAF elimination risks promoting metastasis. **Methods:** In this study, we developed a locally injectable hydrogel based on synergistic dynamic covalent crosslinking (imine and boronate ester bonds), enabling instant gelation, shear thinning, and dual-pH/ROS-responsive degradation. Two types of drug-loaded nanoparticles (NPs), coated with homotypic cell membranes, were incorporated into this hydrogel. In the acidic, reactive oxygen species (ROS)-rich tumor microenvironment (TME), the system responsively releases the antifibrotic drug SIS3 to reprogram CAFs while simultaneously delivering doxorubicin (DOX) specifically to tumor cells. Biological effects were evaluated in vitro using cell cultures and in vivo in mouse models. **Results:** This dynamic hydrogel-based co-delivery system effectively reprograms CAFs, reduces tumor mechanical stress, breaks the fibrotic barrier, and promotes the deep infiltration of chemotherapeutics and immune cells, thereby enhancing the efficacy of chemotherapy. **Conclusions:** This injectable pH/ROS-responsive dynamic covalent hydrogel, loaded with CAF- and cancer cell-targeting NPs, remodels the TME, enhances drug and immune cell penetration, and offers a promising biomaterial-based strategy for cervical cancer treatment.

## 1. Introduction

The tumor microenvironment (TME) plays a critical role in tumor initiation and progression, exerting non-negligible effects on the maintenance of malignant tumor cell phenotypes, immunosuppression, chemoradiotherapy resistance, invasion, and metastasis [1]. The cooperative interaction between tumor cells and stroma leads to tumor occurrence and metastasis within the heterogeneous and dynamic TME network [2], and previous studies have indicated that the fibrous extracellular matrix (ECM) in the TME forms a natural physical barrier that impedes the penetration of drugs and immune cells [3,4]. Cancer-associated fibroblasts (CAFs) are the most predominant stromal cells in the TME and are also the main drivers of ECM stiffening. Aberrant CAF activation leads to excessive deposition and matrix stiffening [5], thereby suppressing immune responses and inducing chemotherapy resistance [6,7]. Although directly depleting CAFs or degrading the ECM might temporarily break the barrier, this approach risks the complete disintegration of the tumor stroma, which paradoxically promotes tumor cell invasion and metastasis [8,9,10,11]. In contrast, a more favorable strategy involves suppressing CAF activation via antifibrotic agents to reprogram CAFs into a quiescent state [11,12].

Transforming growth factor-β (TGF-β) is a key driver that differentiates normal fibroblasts into CAFs by activating various intracellular signaling pathways, such as the TGF-β/Smad pathway [13]. Previous studies have shown that TGFβ1-mediated signaling is involved in CAF activation in breast and colorectal cancers, and that tumor cells can convert stromal fibroblasts into CAFs through paracrine TGF-β signaling in gastric and breast cancers [14,15]. Therefore, targeting TGF-β signaling to reprogram CAFs to a quiescent state, rather than eliminating them, is a strategic approach to reduce the ECM density, alleviate immunosuppression, and enhance chemotherapy efficacy. A promising inhibitor in this context is SIS3 (Specific Inhibitor of Smad3), a selective blocker of the TGF-β/Smad pathway [16,17]. Given that SIS3 requires sustained local exposure to exert its antifibrotic efficacy in the tumor microenvironment, a localized delivery system that prolongs its retention while minimizing systemic clearance would be advantageous. Similarly, co-delivery of chemotherapeutics such as doxorubicin (DOX) requires a platform that can spatiotemporally control the release of multiple agents.

To address these challenges, injectable hydrogels based on dynamic covalent chemistry have emerged as a highly attractive class of biomaterials [18]. Unlike conventional physically crosslinked or permanently chemically crosslinked gels, dynamic covalent hydrogels possess reversible bonds (e.g., imine bonds [19] and boronate ester bonds [20,21]) that combine mechanical stability with stimuli-responsive degradability. These networks can be designed to undergo “instant gelation” upon the mixing of precursor solutions, enabling convenient, minimally invasive injection. Moreover, they exhibit shear-thinning and self-healing properties, which prevent premature leakage and allow the gel to conform to irregular tumor defects. Importantly, because both imine and boronate ester bonds are cleavable under acidic and reactive oxygen species (ROS)-rich conditions, such hydrogels are intrinsically responsive to the TME (low pH [22], high ROS [23]). These unique properties make dynamic covalent hydrogels ideal candidates for the localized, on-demand delivery of therapeutics to solid tumors, including cervical cancer. Intratumoral injections, while not universally applicable across all cancer types, represent a clinically accepted and effective local therapeutic strategy for selected accessible solid tumors (e.g., locally advanced breast cancer, head and neck cancers, or superficial metastases), offering the advantage of bypassing systemic barriers and reducing off-target toxicity [24]. Cervical cancer is readily accessible via the vaginal route for local injection, rendering this approach a particularly advantageous clinical option.

**Figure 1:**
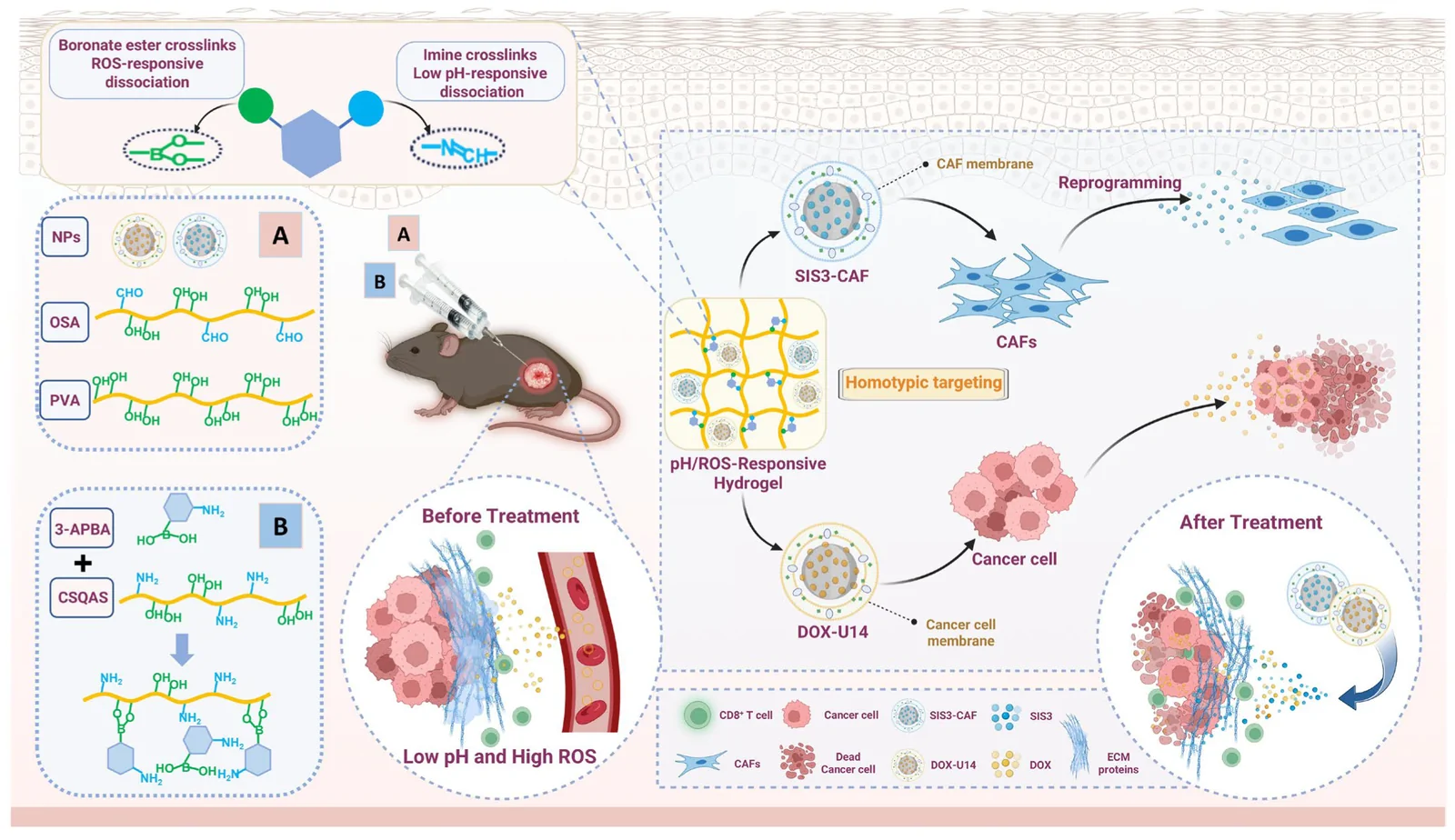
Schematic diagram of pH/ROS-responsive hydrogel for targeted delivery of drug-loaded NPs. Created in BioRender. Chen, Q. (2026) https://BioRender.com/fok90z2.

Despite the availability of radiotherapy, chemotherapy, and immunotherapy, treatment outcomes for cervical cancer remain unsatisfactory because of the development of drug resistance and the poor infiltration of therapeutic agents and immune cells within the fibrotic TME. To address these challenges, we designed a dynamic covalent bond-based (imine and boronate ester bonds) hydrogel system that enables the following: (1) in situ depot formation upon local injection to prolong drug retention; (2) stimuli-triggered drug release in response to the acidic and oxidative TME; and (3) the synergistic delivery of SIS3 to CAFs and DOX to tumor cells through biomimetic targeting. Notably, cell membrane-coated nanoparticles, composed of unique biological cell membrane components and synthetic nanoparticles, represent a promising class of novel nanocarriers. This biomimetic approach enables the replication of natural cellular biological properties and functions, helping nanoparticles to evade immune detection and clearance, thereby reducing off-target effects and toxicity. Surface adhesion molecules on cell membranes facilitate homologous adhesion and homotypic binding, which are commonly observed in specific tumors and can be harnessed for targeted cell delivery, thereby improving internalization and biodistribution [25]. In this study, we designed two distinct types of membrane-coated nanoparticles to achieve the synergistic targeting of different cellular components of the tumor microenvironment. Specifically, two types of drug-loaded nanoparticles (NPs) were constructed from mesoporous silica nanoparticles (MSNs), separately coated with activated CAF cell membranes and murine cervical cancer cell (U14, a commonly used mouse cervical carcinoma cell line) membranes, and then loaded into the hydrogel. The CAF-targeted NPs (SIS3-CAF, short for SIS3-MSN@CAF membrane) deliver SIS3, and the cancer cell-targeted NPs (DOX-U14, short for DOX-MSN@cancer cell membrane) deliver DOX. We expected that the dynamic covalent network would degrade responsively in the weakly acidic, high-ROS TME, releasing both NPs to exert a dual-pronged therapeutic effect. Delivered to CAFs, SIS3 remodels the ECM and disrupts the dense physical barrier, while DOX is specifically delivered to cervical cancer cells (Figure 1). We anticipated that this integrated system would reduce ECM stiffness, alleviate tumor hypoxia, promote the deep penetration of chemotherapeutic agents and immune cells, and enhance chemotherapeutic efficacy while also avoiding the risks associated with direct CAF depletion. With this strategy, we aim to overcome the drug resistance and stromal barriers in cervical cancer, offering a novel localized combination therapeutic approach for advanced or recurrent cervical cancer.

## 2. Materials and Methods

### 2.1. Bioinformatics Analysis

Gene expression data for cervical cancer and normal cervical tissues were obtained from the Gene Expression Omnibus (GEO) repository, including GSE7803, GSE9750, and GSE63514. The sample sizes used in the present analysis were as follows: GSE7803 included 10 normal cervical tissue samples and 21 cervical cancer samples; GSE9750 included 12 normal cervical tissue samples and 26 cervical cancer samples; and GSE63514 included 24 normal cervical tissue samples and 28 cervical cancer samples. Transcriptomic and clinical data for cervical squamous cell carcinoma and endocervical adenocarcinoma (CESC) were obtained from The Cancer Genome Atlas (TCGA) through the Genomic Data Commons portal, with 292 TCGA-CESC samples included in the survival analysis. For GEO microarray datasets, processed expression matrices were downloaded from GEO and analyzed using the normalized signal values provided by the original submitters. Differentially expressed genes (DEGs) between comparison groups were identified using the limma package (version 3.48.3), and genes showing statistically significant changes (*p*-value < 0.05) with consistent directionality in at least two out of the three validation datasets were considered differentially expressed. Subsequently, the correlations between the Actin Alpha 2, Smooth Muscle (ACTA2), Fibronectin 1 (FN1), and Collagen Type X Alpha 1 Chain (COL10A1) gene expression levels were analyzed to elucidate the relationship between fibroblast activation and ECM overexpression. Survival analysis was performed using the Gene Expression Profiling Interactive Analysis 2 (GEPIA2) online platform (http://gepia2.cancer-pku.cn/), which integrates gene expression and clinical data from the TCGA and Genotype-Tissue Expression (GTEx) projects. Overall survival analysis was conducted based on 292 TCGA-CESC samples. Patients were stratified into high- and low-expression groups according to gene expression levels, with the top 20% defined as the high-expression group and the bottom 20% defined as the low-expression group. Patients with intermediate expression levels were not included in the Kaplan–Meier comparison. Kaplan–Meier survival curves were generated, and statistical significance was assessed using the log-rank test.

### 2.2. MSN Synthesis and Characterization

A mixture of 9 mL of cetyltrimethylammonium chloride (CTAC) (Aladdin, Shanghai, China, 25 wt%) and 171 mL of deionized water was stirred at 85 °C to dissolve the CTAC. Then, 0.72 g of triethanolamine (TEA) (Macklin, Shanghai, China), pre-dissolved in 4 mL of deionized water, was added to the solution and stirred for 30 min. Subsequently, 6 mL of tetraethyl orthosilicate (TEOS) (Aladdin) was added, and the reaction was sealed and stirred continuously. After 12 h, the product particles were collected via centrifugation at 12,000 rpm for 40 min, washed three times with HCl/ethanol solution (2%, *v*/*v*), and finally washed three times with ethanol. The dynamic light scattering (DLS) and zeta potential were measured using a Zetasizer Nano ZS90 (Malvern Panalytical, Malvern, UK). The chemical structure of the NPs was examined via FTIR (Fourier Transform Infrared Spectroscopy) (Thermo Fisher Scientific Nicolet iS20, Waltham, MA, USA), and the specific surface area, pore size, and pore volume were determined via a fully automatic surface area and porosity analyzer (BET Micromeritics ASAP 2460, Norcross, GA, USA). The crystal structure of the particles was verified via X-ray diffraction (XRD) (Rigaku SmartLab SE, Akishima, Japan), and the morphology was observed using a scanning electron microscope (SEM) (ZEISS Sigma 360, Oberkochen, Germany) and transmission electron microscope (TEM) (FEI Talos F200x, Hillsboro, OR, USA).

### 2.3. Preparation of Drug-Loaded MSNs

MSNs and DOX were mixed thoroughly in deionized water at a mass ratio of 2:1 (MSNs: 10 mg/mL; DOX: 5 mg/mL) and stirred overnight at room temperature in the dark. MSNs and SIS3 were mixed thoroughly in ethanol at a mass ratio of 2:1 (MSNs: 10 mg/mL; SIS3: 5 mg/mL), followed by ultrasonication for 30 min. The resulting mixture was transferred to a rotary evaporator and evaporated at 40 °C. After evaporation, the product was dried in an oven at 50 °C.

### 2.4. SIS-CAF and DOX-U14 Preparation

CAFs and U14 cells were separately dispersed in a hypotonic buffer (Beyotime, Shanghai, China) containing a protease inhibitor cocktail, and cell membranes were extracted via repeated freeze–thaw cycles. An amount of 50 μL of vesicles derived from CAFs or U14 cells was mixed with 7.5 mg of SIS3-MSNs or DOX-MSNs in 500 μL of ultrapure water, followed by sonication for 5 min in an ice bath. The mixture was then sequentially extruded 15 times through 600 nm and 400 nm polycarbonate porous membranes using an Avanti miniature extruder, and the membrane-coated NPs were collected via centrifugation. After negative staining with phosphotungstic acid, the NP morphology was characterized using a TEM (Hitachi HT7800, Tokyo, Japan).

### 2.5. Synthesis and Characterization of Oxidized Sodium Alginate (OSA)

Sodium alginate (SA) (viscosity: 200 ± 20 mPa·s for 1 wt% aqueous solution at 20 °C) was uniformly dispersed in anhydrous ethanol (concentration: 0.2 g/mL) and magnetically stirred at 40 °C for 30–60 min to obtain a homogeneous suspension without agglomerates. Under light-protected conditions, an aqueous solution of sodium periodate was added dropwise with a molar ratio of sodium periodate to alginate monomer unit of 1:2, and the reaction was stirred at room temperature for 6 h. Then, ethylene glycol (molar ratio to sodium periodate: 1.5:1) was added to terminate the reaction, and stirring continued at room temperature overnight. The reaction suspension was transferred dropwise into 2–3 volumes of stirred anhydrous ethanol to precipitate a white solid, which was collected via suction filtration. The precipitate was redissolved in deionized water, dialyzed against deionized water for 1–2 weeks, and then lyophilized to obtain white cotton-like oxidized sodium alginate (OSA). The aldehyde content of the obtained OSA was determined via hydroxylamine hydrochloride titration, and the relative molecular weight and molecular weight distribution of the OSA were measured via gel permeation chromatography (GPC) (Agilent 1260 Infinity II, Santa Clara, CA, USA).

### 2.6. Synthesis and Characterization of Drug-Loaded Hydrogels

An amount of 0.5 g of polyvinyl alcohol (PVA) 1799 was swollen in 5 mL of water at room temperature with stirring for 30 min and then heated to 95 °C and stirred for 2 h to obtain a transparent solution. The solution was cooled to 80 °C, and 0.2 g of the preprepared OSA was added, followed by continued heating and stirring at 80 °C until dissolution. Then, 5 mL of a pre-sonicated phosphate-buffered saline (PBS) mixture with or without SIS-CAFs and DOX-U14 NPs (200 mg) was added, and the mixture was stirred at room temperature until homogeneous to obtain precursor solution A. An amount of 0.1 g of chitosan quaternary ammonium salt (CSQAS) (Yuanye, Shanghai, China) was swollen in 5 mL of water at room temperature with stirring for 30 min and was then heated to 50 °C and stirred for 2 h. Then, 0.05 g of 3-aminophenylboronic acid (3-APBA) (ACMEC, Shanghai, China) was added, and stirring continued for another 2 h, after which the temperature was raised to 60 °C and stirring continued for 6 h to obtain precursor solution B. For use, equal volumes of precursor solutions A and B were drawn into two 1 mL syringes and placed in a dual-syringe injector (EFL, Suzhou, China) for immediate mixing and injection. For characterization, the hydrogel was injected into a small, transparent sample vial, quickly placed in a 37 °C water bath, and tilted horizontally every 15 s, and the complete gelation time was observed. The chemical structure of the polymers was characterized via FTIR (Thermo Fisher Scientific Nicolet iS20, USA), and the morphology of the freeze-dried hydrogel was observed via an SEM (ZEISS Sigma 360, Germany).

### 2.7. Rheological Testing

Rheological characterization of the hydrogel (Gel) and nanoparticle-loaded hydrogel (Gel-NPs, with an NP concentration of 10 mg/mL) was performed using a rheometer (Anton Paar MCR-301, Graz, Austria) at a constant temperature of 25 °C. The viscosities of both samples as a function of the shear rate were measured over a shear rate range of 0.1–100 s^−1^. Subsequently, strain sweep tests (angular frequency: 10 rad/s; strain range: 0.01–200%) were conducted to determine the linear viscoelastic region and yield point. Within the linear viscoelastic region, a strain of 1% was selected for the frequency sweep tests (angular frequency range: 0.1–100 rad/s), and the storage (G′) and loss (G″) moduli were recorded as functions of the frequency. The self-healing properties of the gels were evaluated using a “three-step intermittent” mode: first, an initial state was established under 2% strain and 10 rad/s for 60 s; then, a destructive large strain (130% for the Gel and 50% for the Gel-NPs, at 10 rad/s) was applied for 30 s; finally, the strain was returned to 2% at 10 rad/s for 60 s to allow for recovery, while the G′ and G″ values were recorded over time.

### 2.8. Swelling Ratio and Degradation Rate Measurement

Hydrogels were placed in centrifuge tubes containing 10 mL of PBS, their initial weights were recorded, and the tubes were then shaken at 37 °C and 100 rpm. At predetermined time points, the hydrogels were removed, blotted dry with filter paper to remove surface moisture, and weighed. When the hydrogels reached their maximum weights and stopped swelling, their weights were measured again to calculate the swelling ratio. For in vitro degradation testing, the initial weight of each hydrogel was recorded, the hydrogels were then placed in PBS (pH 7.4), PBS acidified with HCl (pH 5.5), or PBS containing 1 mM H_2_O_2_ at 37 °C, and the hydrogels were removed and weighed at predetermined time points.

### 2.9. Drug Release

The drug release properties of the hydrogels were determined by loading drug-loaded NPs or hydrogels into dialysis bags (MWCO = 3500). The samples were immersed in PBS at different pH values (containing 0.5% Tween 80, with or without H_2_O_2_) and placed in a shaker at 37 °C, and the release medium was collected and replenished with fresh medium at predetermined time points. The optical density (OD) was measured using an ultraviolet–visible (UV-Vis) spectrophotometer (Thermo Fisher UV-2000, Waltham, MA, USA), and the release rate was calculated.

### 2.10. Cell Culture

The U14 cell line (accession number: FH0931) and the NIH/3T3 cell line (accession number: FH0983) were purchased from FuHeng Biology (Shanghai, China). Both cell lines were sourced from the FuHeng Cell Center database. Both cell lines were cultured in Dulbecco‘s Modified Eagle Medium (DMEM) (high glucose, Gibco, Thermo Fisher Scientific, Waltham, MA, USA) supplemented with 10% fetal bovine serum (FBS) (Gibco) and 1% penicillin–streptomycin (PS) (Solarbio, Beijing, China) at 37 °C in a humidified atmosphere containing 5% CO_2_. To induce cancer-associated fibroblast (CAF) activation, NIH/3T3 cells at 70–80% confluence were treated with 100 ng/mL mouse recombinant transforming growth factor-β1 (TGF-β1) protein (MedChemExpress, Monmouth Junction, NJ, USA) for 24 h. CAF transformation was confirmed via morphological observation and the upregulation of CAF markers (α-SMA) via Western blotting. U14 cells and CAFs were mixed at a ratio of 2:1, seeded at 6000 total cells per well into 96-well U-bottom ultra-low attachment plates (Corning), and cultured at 37 °C for 7 days to establish fibrotic cervical cancer tumor spheroids, with half-medium replacement every 2–3 days. All cell culture-grade reagents were endotoxin-free, and all cell-related experimental manipulations were performed in a clean bench (laminar flow hood).

### 2.11. Cytotoxicity

The MSN or hydrogel cytotoxicity toward U14 and NIH/3T3 cells was evaluated using the Cell Counting Kit-8 (CCK-8, Elabscience, Wuhan, China). U14 and NIH/3T3 cells were seeded into 96-well plates at a density of 5000 cells/well, and MSNs were added to the culture medium at final concentrations of 12.5, 25, 50, and 100 µg/mL. Separately, U14 and NIH/3T3 cells were seeded into 24-well plates at a density of 2 × 10^4^ cells/well, and the prepared hydrogels were added to the medium at final concentrations of 15.625, 31.25, 62.5, and 125 µg/mL. After co-culturing for 24 h and 48 h, the absorbance of each sample was measured at 450 nm using a microplate reader (Thermo Scientific Multiskan SKY, Waltham, MA, USA).

### 2.12. Cellular Uptake

MSNs were loaded with the fluorescent dye coumarin-6 (MCE) to evaluate and statistically compare the cellular uptake of NPs in U14 cells and CAFs. Cells were seeded into 24-well plates at a density of 2 × 10^4^ cells/well and incubated for 6 h. Then, after washing three times with PBS, the cells were fixed with 4% paraformaldehyde (Solarbio) and stained with 4′,6-diamidino-2-phenylindole (DAPI) (Beyotime) for 15 min for nuclear visualization. Images were obtained using a laser scanning confocal microscope (Leica STELLARIS 5, Wetzlar, Germany).

### 2.13. Homotypic Targeting Ability

SIS3-MSN and SIS3-CAF were labeled with coumarin-6 (MCE), while DOX-MSN and DOX-U14 were labeled using DOX autofluorescence (Aladdin). CAFs and U14 cells were co-incubated for 6 h with 1:1 mixtures of SIS3-MSN/SIS3-CAF and DOX MSN/DOX-U14, respectively. Detection was performed using a confocal laser scanning microscope (Leica STELLARIS 5, Wetzlar, Germany), and quantitative analysis was carried out via a spectral flow cytometer (SFLO3, Pukang Medical, Hangzhou, China) and the FlowJo(v10.9) software.

### 2.14. Hemolysis Assay

Fresh blood was collected from the retro-orbital venous plexuses of mice, washed repeatedly with physiological saline, and centrifuged to obtain concentrated red blood cells (RBCs). The RBCs were diluted to a concentration of 5% (*v*/*v*), and equal volumes of hydrogel, PBS, and 0.1% Triton X-100 (Yuanye, China) were incubated with the 5% RBC suspension at 37 °C for 2 h. The OD value of the supernatant at 540 nm was measured using a microplate reader, and the hemolysis rate was calculated.

### 2.15. Wound-Healing Assay

CAFs cultured in 6-well plates were allowed to reach nearly 100% confluence. A scratch was created across the cell monolayer using a pipette tip, and the scratch was observed under a microscope at 0 h and 24 h. The cell migration rate was calculated based on the change in the scratch width at three random positions using ImageJ (version 1.53t).

### 2.16. Transwell Assay

The effect of the drug-loaded hydrogels on the CAF migration ability was evaluated using a Transwell system (NEST). One thousand cells were seeded into the upper chamber of the Transwell system in a serum-free medium, and the hydrogels and medium containing 10% FBS were added to the lower chamber. After 24 h of incubation, cells that had migrated to the bottom surface of the membrane were stained with 1% crystal violet solution, and the stained cells were observed under a microscope and counted.

### 2.17. Live/Dead Staining

U14 cells were seeded into 6-well plates and co-incubated with the hydrogels for 24 h. Because U14 cells are semisuspended, the cells were stained using a Calcein-AM/PI kit (Solarbio), and then all cell samples were collected by grouping, dropped onto glass slides, and mounted, and fluorescent images were captured using a fluorescence microscope (OLYMPUS CKX53, Japan).

### 2.18. Gel Contraction Assay

A rat tail collagen gel contraction assay was performed to evaluate the ECM degradation and CAF fixation effects on the tumor contraction stress. An amount of 5 mg/mL rat tail collagen type I (Solarbio) was added to a 0.1 M NaOH solution, followed immediately by a pre-prepared mixed suspension of U14 cells and CAFs (ratio: 2:1). The mixture was quickly added to a 24-well plate and incubated at 37 °C for 30 min to allow for gelation. The gels were then gently detached so that they floated freely in the culture medium. Gel images were taken after co-incubation with the hydrogels for 48 h.

### 2.19. Animal Experiments

This study evaluated a pH/ROS-responsive hydrogel platform in a U14 mouse cervical cancer model. Twenty-five female C57BL/6 mice were divided into five groups (*n* = 5) and treated after subcutaneous co-inoculation of U14 cells and CAFs. Tumor volume was measured by caliper, hydrogel degradation by fluorescence imaging, and mechanisms (proliferation, apoptosis, fibrosis, angiogenesis, immune infiltration) by histology, IHC, and immunofluorescence. Systemic toxicity was assessed by H&E staining. The SIS3-CAF/DOX-U14@Gel group significantly suppressed tumor growth versus controls (*p* < 0.05) with no obvious toxicity, indicating that this hydrogel system has good efficacy and safety in the U14 model.

All animal protocols were approved by the Biomedical Ethics Committee of Xi’an Jiaotong University Health Science Center (Approval Code: 2021-673, Approval Date: 23 February 2021). No formal protocol was prepared before the start of this study. The experimental approach was developed iteratively based on preliminary observations and was not pre-registered. All mice (wild-type) were purchased from Jiangsu Huachuang Sino Pharmaceutical Technology Co., Ltd., Taizhou, China and were specific pathogen-free. No previous procedures were performed on these animals prior to the start of this experiment. Mice were housed in an SPF facility under controlled conditions (22 ± 2 °C, 50 ± 10% humidity, 12/12 h light/dark cycle) with ad libitum access to food and water. Animals were group-housed (*n* = 5 per cage); no environmental enrichment was provided. A total of 25 mice were utilized. Mice were randomly allocated into five groups using a stratified block randomization method based on baseline body weight, with a block size of 5. Within each block, animals were randomly assigned to the five experimental groups using a computer-generated random number sequence. The sample size (*n* = 5 per group) was chosen according to the standard protocol routinely used in our laboratory for similar tumor models. U14 cells (5 × 10^7^/mL) and CAFs (2.5 × 10^7^/mL) were mixed at a 1:1 volume ratio, and 200 μL of the mixture (containing 5 × 10^6^ U14 cells and 2.5 × 10^6^ CAFs) was subcutaneously injected into the right dorsal flank of each 6-week-old female C57BL/6 mouse (weighing 17–22 g). Body weights and tumor volumes were measured every three days.

Five groups of mice received different treatments: G1: Control (PBS injection); G2: Gel (blank hydrogel injection); G3: SIS3-CAF@Gel (10 mg/mL SIS3-CAF-loaded hydrogel); G4: DOX-U14@Gel (10 mg/mL DOX-U14-loaded hydrogel); G5: SIS3-CAF/DOX-U14@Gel (10 mg/mL SIS3-CAF and 10 mg/mL DOX-U14 co-loaded hydrogel). When the tumor volume reached 100 mm^3^, 100 μL of the respective formulation was injected into the tumor region according to the grouping (DOX: equivalent to 8 mg/kg; SIS3: equivalent to 5 mg/kg). Mice were manually restrained without anesthesia during subcutaneous injections and tumor measurements, as these procedures were brief and minimally invasive. Humane endpoints were predefined (tumor volume > 1000 mm^3^, ulceration, >20% weight loss, or severe distress), and animals meeting these criteria were euthanized. No analgesics were administered as per standard protocol. Health status was monitored daily. No adverse events, expected or unexpected, were observed during the course of the experiment. No animals died unexpectedly, and no signs of severe distress, tumor ulceration, or significant body weight loss were recorded. Mice were euthanized by CO_2_ asphyxiation followed by cervical dislocation. Tumor tissue sections were subjected to hematoxylin and eosin (H&E) staining; terminal deoxynucleotidyl transferase dUTP nick end labeling (TUNEL) fluorescence staining (Yeason 40306ES20); Masson staining (Beyotime C0189S, Shanghai, China); immunohistochemical (IHC) staining for Ki-67 antigen (Ki67), alpha-smooth muscle actin (α-SMA), and cluster of differentiation 31 (CD31); and immunofluorescence staining for cluster of differentiation 8 (CD8) (Invitrogen 740029T, Thermo Fisher Scientific, Waltham, MA, USA). Heart, liver, spleen, lung, and kidney tissues were paraffin-embedded, sectioned, and stained with H&E for observation. A multimodal animal imaging system (AniView100, Biolight Biotechnology, Guangzhou, China) was used to monitor the hydrogel degradation in the mice (days 0, 7, 14, 21, 28), and the fluorescence in the degradation images was quantitatively analyzed. Skin tissue sections after gel injection were stained with H&E for observation.

No specific strategies were employed to minimize potential confounders such as the order of treatments/measurements or animal/cage location. Drug administration, data collection, and data analysis were performed by separate individuals; however, all of them were aware of the group allocation. The inclusion criteria for mice were as follows: (1) good general health with no signs of abnormal behavior or infection; (2) body weight ranging from 17 to 22 g; (3) successful tumor engraftment, defined as stable tumor growth reaching a volume of ≥30 mm^3^ after inoculation; and (4) all mice were female and confirmed non-pregnant. Animals were excluded if they met any of the following criteria: (1) failure to achieve a tumor volume of ≥30 mm^3^ after tumor inoculation; (2) non-treatment-related death or accidental infection during the experiment; (3) tumor volume exceeding 1000 mm^3^. No criteria were established a priori for the exclusion of individual data points during the analysis. These criteria follow the standard protocol routinely used in our laboratory for similar tumor models. No animals or data points were excluded from the analysis. All 25 mice successfully completed the experiment and were included in the final statistical analysis. The primary outcome measure used to determine the sample size was tumor volume, which was assessed by caliper measurement every 3 days and calculated as V = (L × W^2^)/2. All experimental results were presented as means ± SDs. Quantitative analysis of IHC and IF images was performed using ImageJ software (National Institutes of Health, Bethesda, MD, USA). Statistical analyses were performed using GraphPad Prism 9.0. For repeated-measures data (tumor volume and body weight over time), two-way repeated-measures ANOVA followed by Bonferroni’s post hoc test was used for multiple-group comparisons. For endpoint data (final tumor weight, histological indices, and quantitative IHC/IF results), one-way ANOVA followed by Tukey’s post hoc test was used for multiple-group comparisons. Normality and homogeneity of variances were assessed using Shapiro–Wilk and Levene’s tests. Data that did not meet these assumptions were log-transformed; if normality remained unachieved, non-parametric Kruskal–Wallis with Dunn’s post hoc test was applied instead of ANOVA. A *p*-value < 0.05 was considered statistically significant.

### 2.20. IHC and Tissue Immunofluorescence (IF) Staining

Tumor sections were stained with anti-α-SMA (Proteintech 14395-1-AP, Rosemont, IL, USA), anti-Ki67 (Proteintech 27309 1 AP, Rosemont, IL, USA), anti-CD31 (Invitrogen PA5 143217, Thermo Fisher Scientific, Waltham, MA, USA), anti-CD8 (Invitrogen 740029T, Thermo Fisher Scientific, Waltham, MA, USA), and horseradish peroxidase (HRP)-conjugated (Servicebio GB 23303, Wuhan, China) secondary antibodies.

### 2.21. Cellular Immunofluorescence Staining

NIH/3T3 cells or CAFs were cultured on coverslips and subjected to different treatments. Primary antibody anti-α-SMA (1:2000, YA3468, MCE) was incubated overnight. After three washes with PBS, cells were incubated with fluorescein isothiocyanate (FITC)-labeled goat anti-mouse secondary antibody (Servicebio GB25303, Wuhan, China) or Cyanine 3 (Cy3)-labeled secondary antibody (Servicebio GB21301, Wuhan, China) for 1 h.

### 2.22. Western Blot Analysis

Protein levels of SMAD family member 3 (SMAD3), phosphorylated SMAD3 (p- SMAD3), and α-SMA in NIH/3T3 cells or CAFs were analyzed. The primary antibodies used were anti-α-SMA (1:5000, MCE YA3468), anti-p-SMAD3 (1:1000, MCE HY P80854), anti-SMAD3 (1:1000, MCE HY P80325), and anti-GAPDH (1:3000, Servicebio GB15004-100), incubated overnight at 4 °C, followed by incubation with HRP-conjugated goat anti-rabbit secondary antibody (1:3000, Servicebio GB23303) for 2 h at room temperature. Imaging was performed using a chemiluminescence imager (Servicebio SCG W3000). To evaluate the distribution of characteristic protein bands in cell membrane-coated nanoparticles, sodium dodecyl sulfate-polyacrylamide gel electrophoresis (SDS-PAGE) was performed. After electrophoresis, the gel was stained with Coomassie blue to visualize the protein bands. Furthermore, Western blotting was carried out to assess key proteins in the experimental samples, with primary antibodies targeting Na^+^/K^+^-ATPase (1:1000, Servicebio GB15400-100), GAPDH (1:3000, Servicebio GB15004-100), cytochrome C (1:1000, Servicebio GB15080-100), and histone H3 (1:1000, Servicebio GB15102-100).

### 2.23. Statistical Analysis

All data were obtained from at least three independent experiments and are presented as means ± SDs. Statistical analyses were performed using the GraphPad Prism (v9.0.0) software. One-way analysis of variance (one-way ANOVA) followed by Tukey’s post hoc test was used for multiple-group comparisons, and Student’s *t*-test was used for two-group comparisons. A *p* value < 0.05 was considered statistically significant (significance levels in figures are indicated as NS for no significant difference, * *p* < 0.05, ** *p* < 0.01, *** *p* < 0.001, and **** *p* < 0.0001).

## 3. Results

### 3.1. Bioinformatics Analysis of CAF- and ECM-Related Signatures in Cervical Cancer

**Figure 2:**
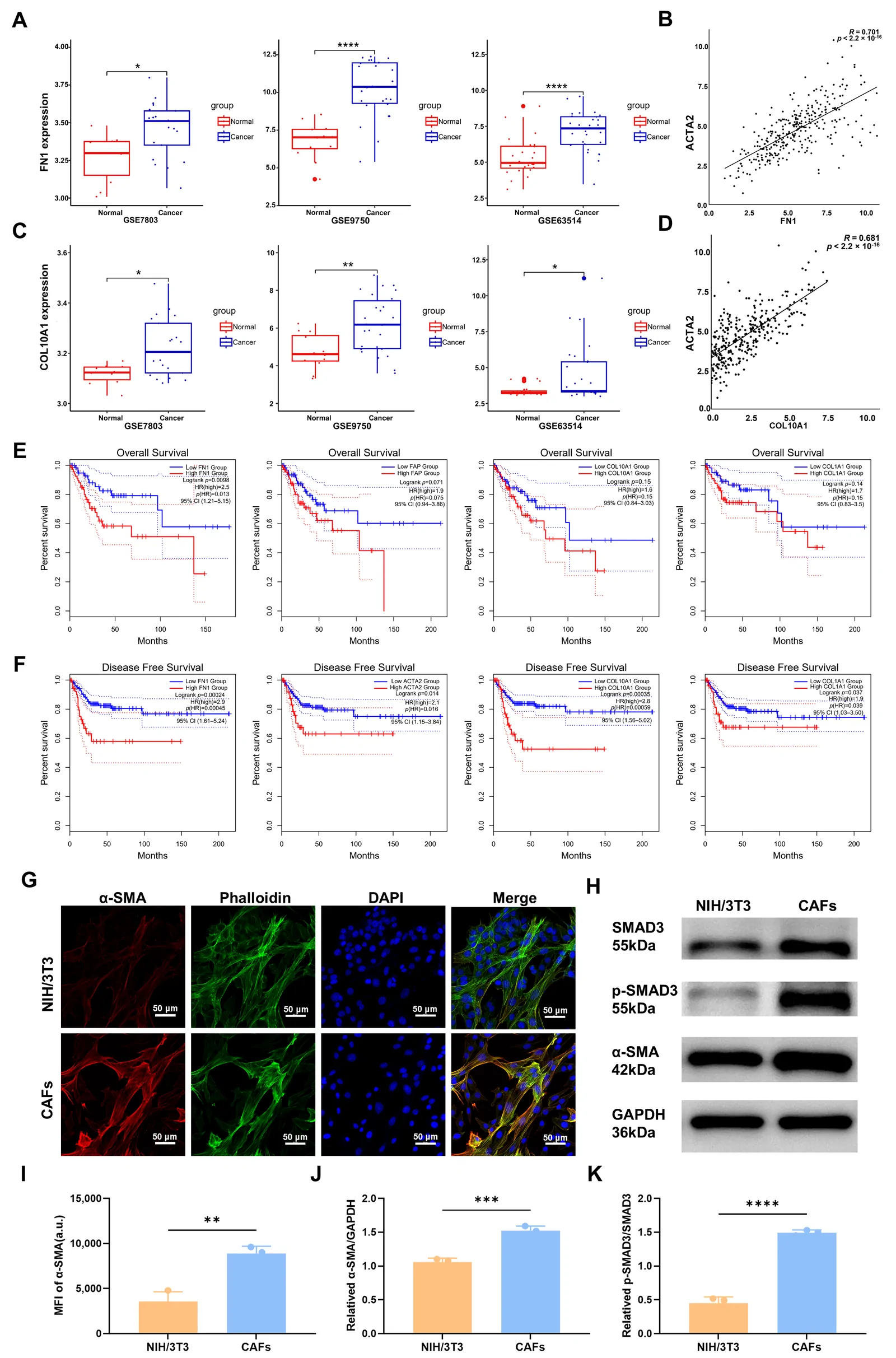
Bioinformatics analysis and experimental validation of cervical cancer fibrosis. Differential expression analysis of (**A**) FN and (**C**) COL10 in cervical cancer. Correlation analysis of (**B**) FN and (**D**) COL10A1 with ACTA2 gene expression in cervical cancer patients from GDC TCGA database; 0.6 < *R* ≤ 0.8 indicates strong positive correlation. (**E**,**F**) Survival analysis of cervical cancer patients based on expression of ECM-related genes (FN1, FAP, COL10A1, COL1A1, ACTA2). The dotted lines represent the 95% confidence intervals for the survival estimates. (**G**,**I**) Immunofluorescence and statistical analysis of α-SMA protein and cytoskeleton. Scale bars: 50 μm. (**H**,**J**,**K**) Western blot images and statistical analysis of SMAD3, p-SMAD3, and α-SMA in NIH/3T3 cells and CAFs. Data are shown as mean ± SD (*n* = 3). Statistical significance was determined by two-tailed Student’s *t*-test. * *p* < 0.05; ** *p* < 0.01; *** *p* < 0.001; **** *p* < 0.0001.

Hyperactivated fibroblasts within tumors express abnormal ECM proteins, such as fibronectin (FN) and collagen (COL). By analyzing clinical cervical cancer patients through bioinformatics, we compared the RNA expression profiles between tumor and normal tissues across multiple datasets from the GEO repository. The results revealed FN and COL10A1 upregulation in tumor tissues compared to normal controls (Figure 2A,C). We then examined the correlation between ACTA2 and FN1/COL10A1 expression in the TCGA data. Interestingly, we found that ACTA2 was positively correlated with both FN1 and COL10A1 in tumor tissues (Figure 2B,D). The protein α-SMA, regulated by the ACTA2 gene, is a key marker of CAFs [26]. Furthermore, existing clinical sample studies have shown that the intensity of α-SMA immunostaining in cervical cancer is 5.2 times higher than that in normal tissues [27]. Therefore, we proceeded to examine publicly available datasets to assess whether CAF activation and the presence of ECM components such as collagen are associated with the survival rate of cervical cancer patients. Cervical cancer patients with higher FN1 expression had reduced overall survival and disease-free survival (Figure 2E,F), and those with higher ACTA2, COL10A1, and Collagen type I alpha 1 chain (COL1A1) expression had reduced disease-free survival. Although not reaching statistical significance, cervical cancer patients with high Fibroblast Activation Protein (FAP), COL10A1, and COL1A1 expression showed a trend of lower overall survival rates (Figure 2E). These results once again confirm our analysis and hypothesis that overactivated CAFs in cervical cancer greatly enhance ECM accumulation, and this upregulation leads to dense tumor stroma and increases mechanical pressure within the TME, which, in turn, severely constricts the tumor vasculature, resulting in a hypoxic state in the tumor and surrounding tissues and hindering drug penetration and immune cell infiltration. This may be one of the main reasons for chemotherapy failure in cervical cancer.

Tumor-secreted TGF-β is a major catalyst for fibroblast activation. Previous studies have confirmed that adding TGF-β1 recombinant protein to a standard culture medium can convert mouse embryonic fibroblasts (NIH/3T3) into CAFs [12]. We also successfully converted NIH/3T3 cells into CAFs by supplementing the culture medium with an additional 100 ng/mL of TGF-β1 protein. Immunofluorescence staining observed using confocal laser scanning microscopy confirmed the upregulation of the α-SMA protein (green fluorescence) in CAFs (Figure 2G,I). Western blot and quantitative analysis further confirmed this conversion, showing a significantly increased p-SMAD3/SMAD3 ratio in CAFs (Figure 2H,K), indicating the successful activation of the TGF-β/Smad3 signaling pathway via the TGF-β1 protein, leading to elevated α-SMA protein levels in CAFs (Figure 2H,J). These results indicate that cytokines secreted by tumor cells promote CAF activation and ECM fibrosis, with TGF-β playing a critical role. We speculate that the targeted inhibition of CAF activation induced by tumor-secreted TGF-β may effectively reduce tumor tissue fibrosis and remodel the ECM, thereby overcoming the barriers to drug delivery and immune cell infiltration in cervical cancer and restoring responsiveness to chemotherapy.

### 3.2. MSN Preparation and Characterization

**Figure 3:**
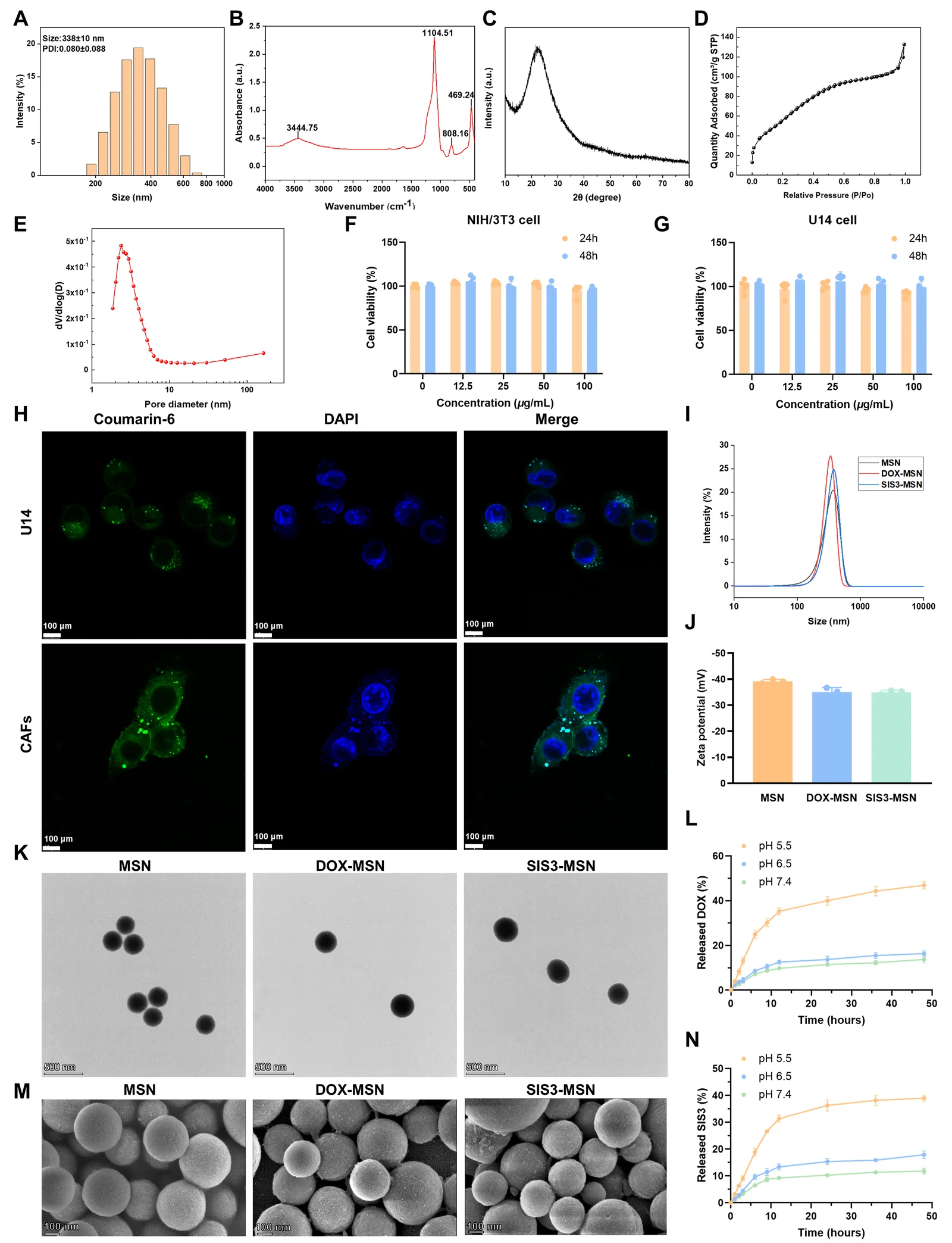
MSN preparation and characterization. (**A**) Hydrodynamic diameters of MSNs. (**B**) FTIR spectra of MSNs. (**C**) XRD pattern of MSNs. (**D**) N_2_ adsorption–desorption isotherm plot of MSNs. (**E**) BJH pore size distribution plot of MSNs. (**F**,**G**) Cell viability of NIH/3T3 and U14 cells after incubation with MSNs for 24 or 48 h, as determined via CCK-8 assay. (**H**) Uptake images of MSNs in U14 cells and CAFs. Scale bars: 100 μm (**I**) Hydrodynamic diameters, (**J**) zeta potentials, (**K**) TEM images (Scale bars: 500 nm), and (**M**) SEM images of MSNs, DOX-MSNs, and SIS3-MSNs (Scale bars: 100 nm). (**L**,**N**) Cumulative release profiles of DOX and SIS3 from MSNs in PBS under different pH conditions. Data are shown as mean ± SD (*n* = 3). Statistical significance was determined by One-way ANOVA with Tukey’s post hoc test.

MSNs prepared via a modified sol–gel method [28] exhibited a particle size of 338 ± 10 nm and a polydispersity index (PDI) of 0.080 ± 0.088, as determined via DLS (Figure 3A). In the MSN FTIR spectrum, distinct absorption peaks appear at 469.24 cm^−1^, 808.16 cm^−1^, and 1104.51 cm^−1^ (Figure 3B). The peaks at 469.24 cm^−1^ and 808.16 cm^−1^ correspond to the bending and stretching vibrations of Si-O-Si bonds, respectively, while the peak at 1104.51 cm^−1^ is attributed to the asymmetric stretching vibration of Si-O-Si [29]. The weaker absorption peak observed at 3444.75 cm^−1^ is due to the O-H stretching vibration of water molecules adsorbed on the silica surface, which may have resulted from the absorption of a small amount of water on the sample surface during exposure to air. The FTIR spectrum was consistent with the standard silica spectrum, indicating that these MSNs possess typical silica characteristics. XRD results showed no sharp peaks characteristic of crystalline substances. Instead, a nearly symmetric and relatively broad amorphous diffraction peak was observed at 2θ = 22.580°, indicating that these MSNs have an amorphous, non-crystalline structure (Figure 3C). The average specific surface area of the MSNs was 231.11 m^2^/g, the average pore volume was 0.205 cm^3^/g, and the average pore diameter was 3.8 nm (Figure 3D,E). These values are in accordance with the definition of mesoporous materials by the International Union of Pure and Applied Chemistry (IUPAC), which specifies that mesoporous materials are those with pore diameters between 2 and 50 nm.

The MSN cytotoxicity was evaluated using the CCK-8 assay. MSNs were added to the culture medium at final concentrations of 12.5, 25, 50, and 100 µg/mL and then co-cultured with NIH/3T3 (Figure 3F) and U14 (Figure 3G) cells. Cell viability was assessed after 24 h and 48 h of co-culturing. Even at a concentration as high as 100 µg/mL, MSNs exhibited negligible cytotoxicity in both cell types, indicating good biocompatibility. Furthermore, we observed the endocytosis of MSNs in U14 cells and CAFs. MSNs were labeled with coumarin-6 and added to the culture medium. After 6 h of incubation, confocal laser scanning microscopy revealed the efficient uptake of MSNs by both cell types (Figure 3H).

Previous studies have shown that SIS3 can reprogram activated CAFs into a quiescent state without compromising cell viability [30]. Although the inhibitory effect of SIS3 on the TGF-β/Smad pathway has been demonstrated, SIS3 faces several pharmacokinetic challenges, including low water solubility, low bioavailability, and potential nephrotoxicity at high doses, which largely limit its clinical application [31]. Typically, SIS3 is first dissolved in an organic solvent such as dimethyl sulfoxide (DMSO) and then diluted into aqueous buffers, which may lead to additional toxicity and a higher risk of vascular embolism caused by drug precipitation [32]. Meanwhile, the bioavailability of small-molecule drugs is also limited by their relatively short half-lives due to hepatic metabolic and renal clearance, which results from their small size [33]. To overcome these limitations, nanoparticle-based drug delivery systems have been employed to improve the aqueous stability of nanoparticle-based drugs and enhance the EPR effect in tumor tissues, thereby increasing tumor targeting efficacy [34] and prolonging the circulation half-life while reducing toxicity [35,36]. Thus, employing MSNs as nanocarriers for SIS3 enables a notable improvement in the SIS3 bioavailability, biodistribution, and biocompatibility. In addition, a notable advance in the application of hydrogels for cancer therapy involves loading other types of nanostructures into the hydrogel network to maximize the delivery process and promote tumor suppression. This strategy has also been widely employed for DOX delivery to improve therapeutic outcomes in cancer treatment [37].

After loading with DOX and SIS3, the MSN, DOX-MSN, and SIS3-MSN morphologies were observed via TEM and SEM, respectively (Figure 3K,M). MSN, DOX-MSN, and SIS3-MSN were all approximately spherical, well dispersed, and showed no significant aggregation, and the average particle diameters measured via electron microscopy were 246 ± 61 nm, 283 ± 80 nm, and 257 ± 72 nm, respectively. The maximum loading capacities (LCs) of DOX-MSNs for DOX and SIS3-MSNs for SIS3 when the drug-to-carrier feeding ratio was 2:1 (*w*/*w*) were 28.03 ± 2.17% and 21.26 ± 3.51%, respectively. We selected the 1:2 feeding ratio for the subsequent in vitro and in vivo studies to balance high drug loading and efficient drug utilization. For DOX-MSNs, the LC was 16.17 ± 1.52% with an encapsulation efficiency (EE) of 38.58 ± 4.33%, and for SIS3-MSNs, the LC was 10.54 ± 1.09% with an EE of 23.56 ± 2.73%. After loading DOX and SIS3, the hydrodynamic diameters measured via DLS were 376 ± 8 nm and 362 ± 5 nm, respectively (Figure 3I), with PDI values of 0.249 ± 0.041 and 0.200 ± 0.066, respectively. The hydrodynamic diameters of MSNs increased after DOX and SIS3 loading, and zeta potential measurements showed that the surface charge also increased after DOX and SIS3 loading (−39.13 ± 0.75 mV, −35.07 ± 1.70 mV, and −35.03 ± 0.74 mV) (Figure 3J). DOX-MSNs exhibited pH-responsive release, with the slow release of DOX from MSNs under neutral pH conditions but a greatly accelerated release rate under acidic conditions. SIS3-MSNs also showed a similar pH-dependent drug release profile (Figure 3L,N).

### 3.3. Homotypic Targeting of SIS3-CAF and DOX-U14

**Figure 4:**
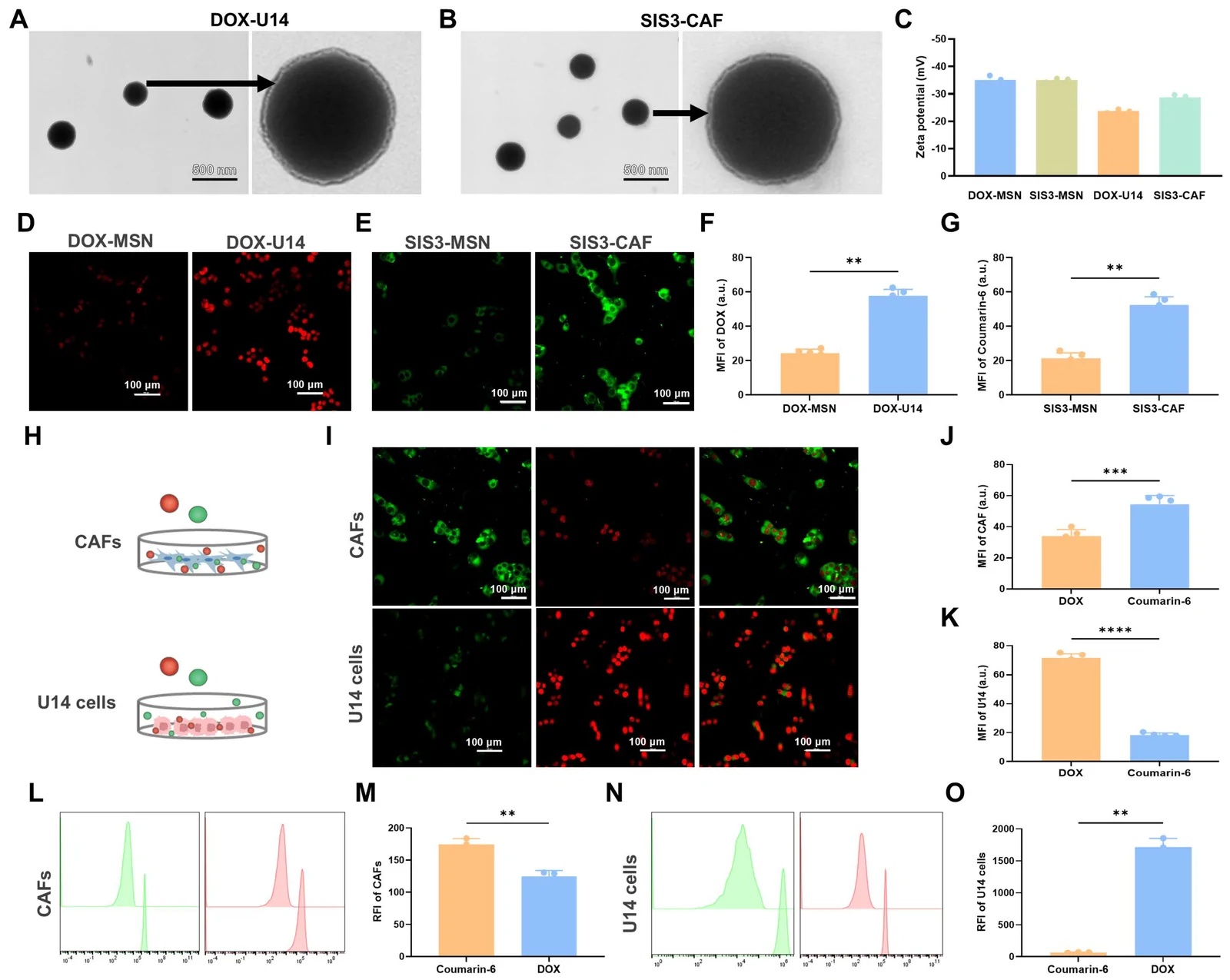
DOX-U14 and SIS3-CAF characterization. Representative TEM images of (**A**) DOX-U14 and (**B**) SIS3-CAFs. Scale bars: 500 nm. (**C**) Zeta potentials of indicated NPs. (**D**,**F**) Confocal fluorescence imaging and statistical analysis of U14 cells after incubation with DOX-MSNs or DOX-U14. Scale bars: 100 μm. (**E**,**G**) Confocal fluorescence imaging and statistical analysis of CAFs after incubation with coumarin-6-labeled SIS3-MSNs or SIS3-CAFs. Scale bars: 100 μm. (**H**,**I**–**K**) Homotypic targeting ability of DOX-U14 and SIS3-CAFs. Confocal fluorescence imaging and statistical analysis of U14 cells and CAFs after incubation with 1:1 mixture of DOX-U14 and SIS3-CAFs. Created in BioRender. Chen, Q. (2026) https://BioRender.com/u786fi7. Green represents SIS3-CAF, Red represents DOX-U14. (**L**–**O**) Flow cytometry MFI of CAFs and U14 cells after incubation with 1:1 mixture of DOX-U14 and SIS3-CAFs. Green represents SIS3-CAF, Red represents DOX-U14. Data are shown as mean ± SD (*n* = 3). Statistical significance was determined by two-tailed Stu-dent’s *t*-test and One-way ANOVA with Tukey’s post hoc test. ** *p* < 0.01; *** *p* < 0.001; **** *p* < 0.0001.

As described above, we converted NIH/3T3 fibroblasts into CAFs via TGF-β1 supplementation, which resulted in a stellate morphology and the acquisition of the biomarker α-SMA. To achieve homotypic targeting delivery, we further coated the MSNs with CAF membranes and cancer cell membranes to form two types of NPs. Successful coating with CAF membranes and U14 cell membranes was observed via a TEM, revealing a clear core/shell structure (Figure 4A,B). After membrane coating, the surface charge increased, as measured via the zeta potential, with values of −23.67 ± 0.70 mV for DOX-U14 and −28.73 ± 1.12 mV for SIS3-CAFs (Figure 4C). To characterize the protein composition of the cell membrane-coated MSNs, we performed SDS-PAGE analysis. As depicted in Figure S1A, the protein banding patterns of DOX-U14 and SIS3-CAF were comparable to those of the corresponding native cell membranes. Furthermore, Western blot analysis (Figure S1B,C) demonstrated that intracellular markers, including GAPDH (cytoplasmic marker), cytochrome C (mitochondrial marker), and histone H3 (nuclear marker), were barely detectable in DOX-U14 and SIS3-CAF. Notably, Na^+^/K^+^-ATPase was retained in both DOX-U14 and SIS3-CAF relative to intact cells, indicating that the membrane-associated proteins were well preserved during the coating process. In addition, as shown in Figure S2A,B, we evaluated the stability of the nanoparticles. The nanoparticles maintained a stable particle size in PBS at 4 °C for 5 days, and remained stable particle size for 48 h in DMEM containing 20% FBS, indicating that the prepared nanoparticles possessed good stability. We fluorescently labeled SIS3-MSNs and SIS3-CAFs with coumarin-6 and used the intrinsic fluorescence of DOX to monitor the nanoparticle endocytosis via confocal fluorescence imaging and flow cytometry. Compared with uncoated NPs (SIS3-MSNs and DOX-MSNs), membrane coating significantly enhanced the internalization of SIS3-CAFs by CAFs and that of DOX-U14 by U14 cells (Figure 4D–G). Next, we investigated whether CAFs and cancer cells preferentially uptake their corresponding fluorescent particles. To test this concept, CAFs and U14 cells were each incubated with a 1:1 mixture of SIS3-CAFs and DOX-U14 (Figure 4H) [12]. Confocal fluorescence microscopy showed that CAFs preferentially took up SIS3-CAFs (Figure 4I,J), while the uptake of DOX-U14 was dominant in U14 cells (Figure 4I,K). These results were further confirmed via flow cytometry, indicating that these two types of NPs can be differentially taken up by CAFs and cancer cells through homotypic targeting (Figure 4L–O). Based on these results, we observed that these MSNs, coated with different cell mem-branes, showed selective uptake towards their respective target cells during the co-delivery of DOX and SIS3.

### 3.4. Design and Synthesis of Stimuli-Responsive Hydrogels

**Figure 5:**
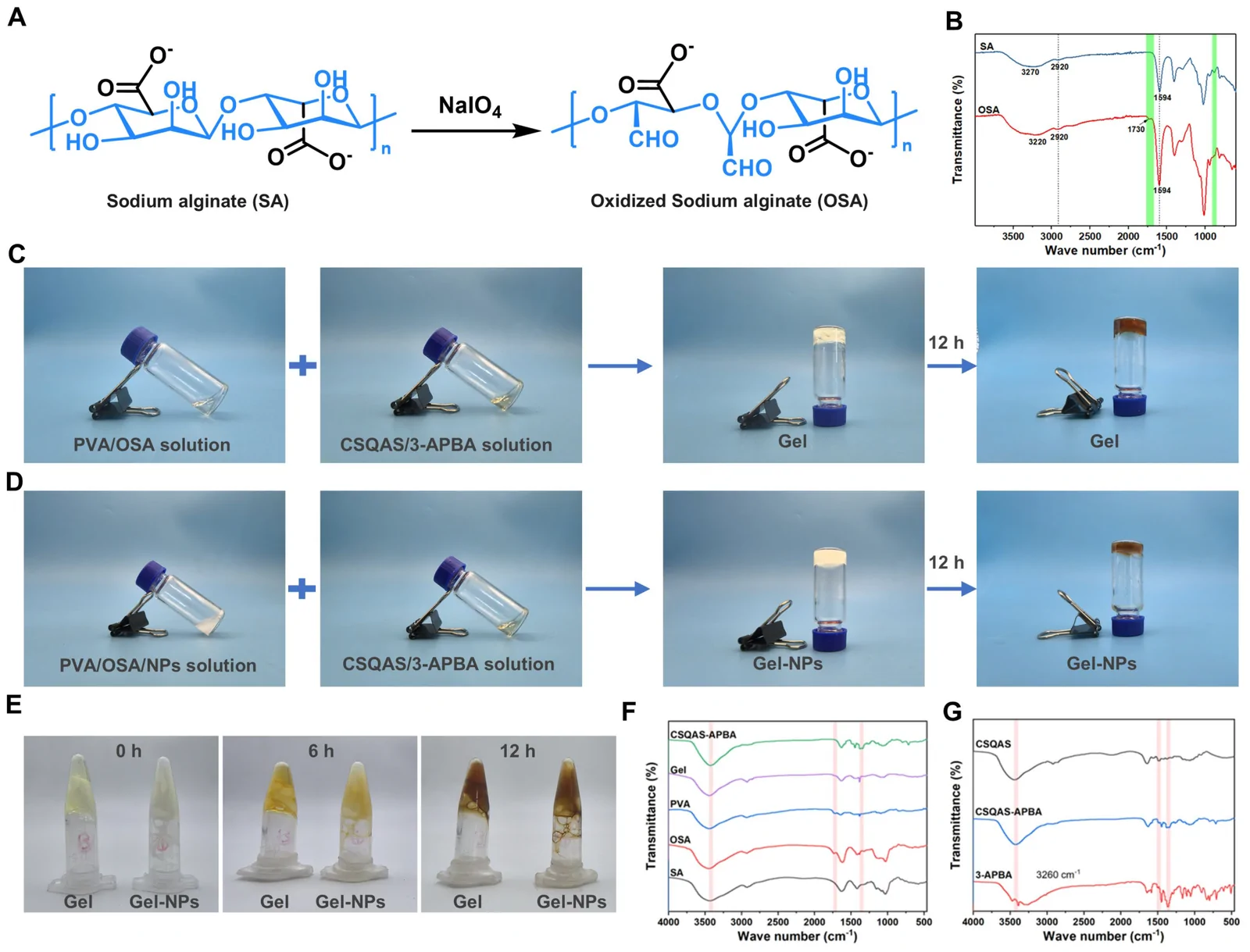
Design and synthesis of stimuli-responsive hydrogels. (**A**) Schematic diagram of OSA synthesis reaction. (**B**) FTIR spectra of SA and OSA. (**C**,**D**) Photographs of precursor solution A (prepared from PVA/OSA solution with and without NPs) uniformly mixed with equal volume of precursor solution B (prepared from CSQAS/3-APBA solution) to form hydrogel. (**E**) Color change photographs of hydrogels with and without NPs. (**F**,**G**) FTIR spectra of SA, OSA, PVA, CSQAS, 3-APBA, CSQAS-APBA, and Gel.

Stimuli-responsive hydrogels, with their excellent biocompatibility, stability, drug-loading capacity, and responsiveness to environmental stimuli, have become outstanding carriers for efficient and intelligent antitumor drug delivery [37], and various therapeutic agents, such as chemotherapeutic drugs, proteins, photosensitizers, siRNA, and functional nucleic acids, can be loaded and delivered via hydrogels. In view of the unique mildly acidic and high ROS microenvironment of tumors, we designed and prepared hydrogels that can be injected to form in situ at the tumor site and achieve the sustained and controlled release of nanomedicines under low-pH and high-ROS stimulation. To this end, we developed a hydrogel system constructed using reversible C=N crosslinks (imine bonds, pH-responsive) and boronate ester bonds (ROS-responsive). By adjusting the ratio of SA to sodium periodate, we obtained an OSA with a certain degree of aldehyde functionalization (Figure 5A). The aldehyde group content of this OSA, determined via hydroxylamine hydrochloride titration, was 6.5 mmol/g, corresponding to a 64.35% oxidation degree. The relative molecular weight of the OSA was measured via GPC as 154 kDa, with a dispersity (DPI) of 1.29. The successful synthesis of this OSA was further confirmed by comparing the SA and OSA FTIR spectra (Figure 5B). The characteristic peaks of the SA and OSA at 3270–3220 cm^−1^, 2920 cm^−1^, and 1594 cm^−1^ correspond to the O-H stretching vibration, C-H stretching vibration, and asymmetric stretching vibration of -COO- on the molecular chains, respectively. These three characteristic peaks were present in both the SA and OSA. Compared with that of the SA, the O-H peak of the OSA showed a slight redshift and decrease in intensity. Moreover, a new characteristic absorption peak appeared at 1730 cm^−1^ in the OSA, corresponding to the stretching vibration of aldehyde (C=O), indicating that the OSA indeed possessed fewer hydroxyl groups and newly formed aldehyde groups compared to the SA. The characteristic absorption peak of the SA at 880 cm^−1^, attributed to the C-O-C stretching vibration of the glycosidic bond, became sharper in the OSA, which might be due to partial breakage caused by the non-specific oxidative cleavage of the glycosidic bonds in the SA via NaIO4, combined with the overlapping characteristic peak of the hemiacetal structure formed by the condensation reaction between the aldehyde groups of the OSA and some of the vicinal dihydroxy groups. These FTIR data confirmed that NaIO4 successfully oxidized some of the hydroxyl groups on the SA molecular chains into aldehyde groups, producing an OSA with an aldehyde structure.

Precursor solution A (PVA/OSA solution with or without NPs) was uniformly mixed with an equal volume of precursor solution B (CSQAS/3-APBA solution). The gelation time of the Gel was determined to be 1.45 ± 0.19 min, whereas that of the Gel-NPs was 2.05 ± 0.29 min, indicating a slight prolongation upon nanoparticle loading (Figure 5C,D). In our experiments, the minimum gelation time of the Gel was 1.25 min, whereas that of the Gel-NPs was 1.75 min. These gelation times represent a favorable balance for injectable hydrogel applications, as they are sufficiently short to ensure rapid in situ gelation and retention at the injection site, thereby preventing precursor dispersion before network formation, but not so short as to cause premature solidification during injection, which could otherwise lead to needle clogging. The slightly prolonged gelation of the Gel-NPs compared with that of the Gel is attributed to the NPs’ incorporation, which may partially hinder polymer crosslinking; however, this difference does not compromise injectability, as both formulations were smoothly delivered through a 25 G needle without clogging. Upon incubation at 37 °C, the hydrogel gradually changed from pale yellow to tan within 12 h (Figure 5E), which is attributed to the further condensation of residual aldehyde groups from the OSA with amino groups of CSQAS, forming conjugated Schiff-base products. To evaluate the impact, we measured the rheological properties and swelling ratios at 12 h in the subsequent experiments. The results indicated that the color alteration did not significantly affect the bulk mechanical or water uptake properties, although it suggested a slight increase in the crosslinking density over time.

The synthesized hydrogels were further characterized via FTIR [38]. By comparing the FTIR spectra, it was found that the characteristic peak of the aldehyde group (-CHO) at 1730 cm^−1^, corresponding to the C=O stretching vibration, which appeared in the OSA compared to the SA, was significantly weakened or even disappeared in the Gel (Figure 5F), demonstrating that the aldehyde groups of the OSA underwent a Schiff-base reaction with the amino groups of CSQAS to form imine bond crosslinking. Moreover, compared with CSQAS-APBA, the O-H stretching vibration peak of the boronic acid group in 3-APBA at 3260 cm^−1^ almost disappeared, and the characteristic doublet peaks of the amino group in 3-APBA at 3470 cm^−1^ and 3390 cm^−1^ were significantly diminished (Figure 5G). Meanwhile, the B-O stretching vibration peak of 3-APBA at approximately 1360 cm^−1^ was partially blue-shifted to the range of 1360–1390 cm^−1^ in the CSQAS-APBA spectrum. Furthermore, the characteristic peak of the quaternary ammonium group (1480 cm^−1^) and the hydroxyl group peak (3430 cm^−1^) of CSQAS remained present in the CSQAS-APBA spectrum with almost no shift in position (Figure 5G). These favorable results demonstrated that 3-APBA was successfully grafted onto CSQAS via boronate ester bonds to form the target polymer CSQAS-APBA. In addition, the broad O-H stretching vibration peak of PVA and the vicinal diol groups (-CH(OH)-CH(OH)-) in the OSA at 3200–3400 cm^−1^ became narrower and decreased in intensity in the Gel. The peak at 1390 cm^−1^ corresponding to the B-OH stretching vibration of the phenylboronic acid group (-B(OH)_2_) of APBA in CSQAS-APBA, as well as the shoulder peak at 3200–3400 cm^−1^ arising from the overlap of B-OH and hydroxyl groups, were attenuated in the Gel (Figure 5F). These observations indicate that the vicinal diol groups of the PVA and OSA underwent an ester exchange reaction with the grafted phenylboronic acid groups of CSQAS-APBA to form boronate ester bond crosslinking.

In summary, the Gel forms through synergistic crosslinking involving imine bonds (from aldehyde groups of OSA and amino groups of CSQAS) and boronate ester bonds (from vicinal diols of PVA/OSA and phenylboronic acid groups of CSQAS-APBA), and the interweaving of these two types of dynamic covalent bonds constructs a three-dimensional network, ultimately achieving the transformation from solution to hydrogel.

### 3.5. Characterization of Stimuli-Responsive Hydrogels

**Figure 6:**
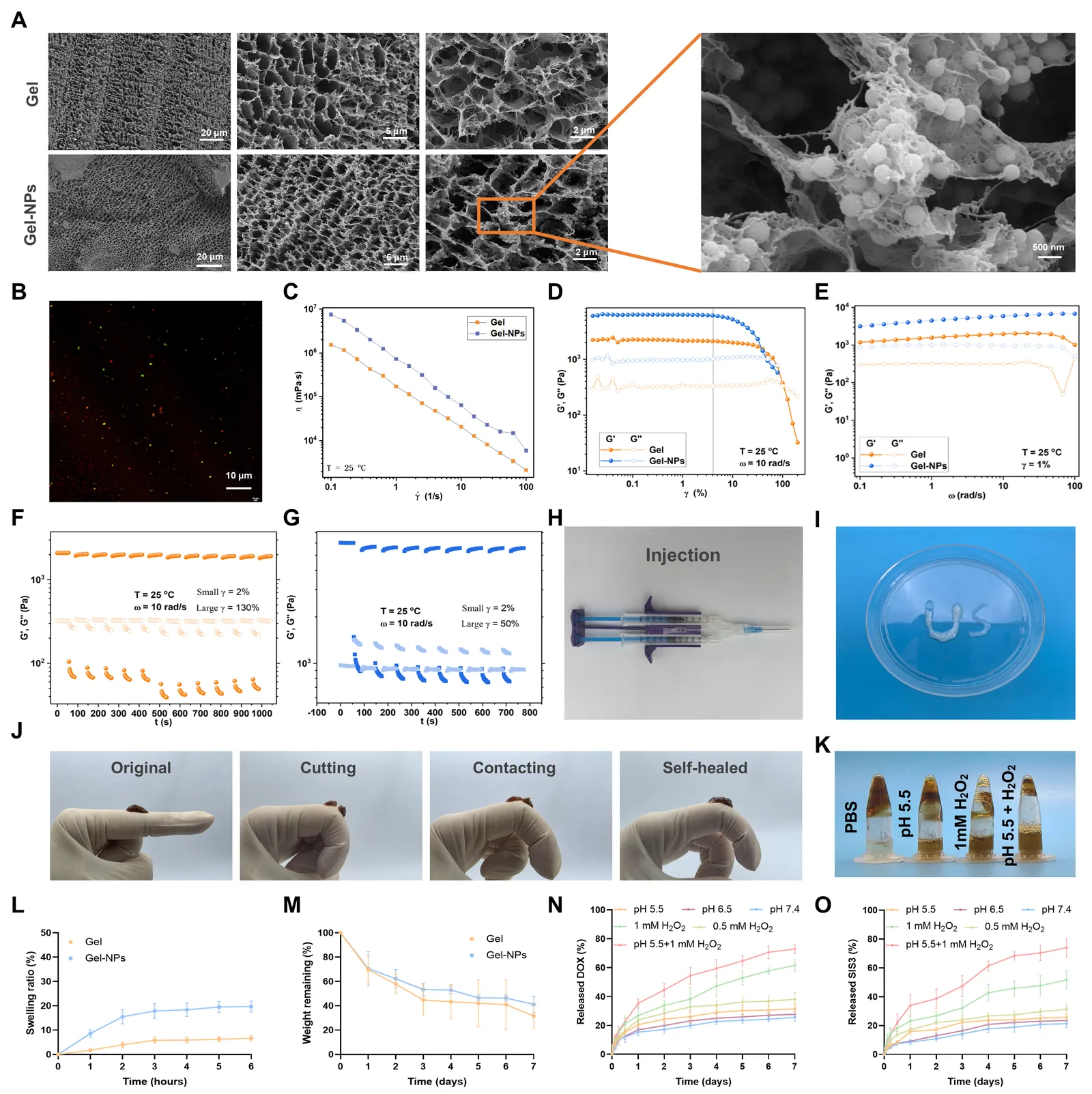
Characterization of stimuli-responsive hydrogels. (**A**) Representative SEM images of freeze-dried hydrogels with and without NPs. Scale bars from left to right: 20 μm, 5 μm, 2 μm, and 500 nm. (**B**) Representative confocal fluorescence imaging of SIS3-CAF/DOX-U14@Gel. Scale bars: 10 μm. (**C**) Viscosity of Gel and Gel-NPs as a function of shear rate. (**D**) Rheological strain sweep results of Gel and Gel-NPs. (**E**) Rheological frequency sweep results of Gel and Gel-NPs. (**F**,**G**) Recovery of G′ and G″ after applying large strain (130% for Gel; 50% for Gel-NPs) disrupting hydrogel structure. (**H**,**I**) Uniform and continuous co-injection of equal volumes of preprepared precursor solutions A and B through a double-barrel syringe to form letters “U” and “S”. (**J**) Macroscopic photographs showing the self-healing property of hydrogel. (**K**) Macroscopic observation of pH/ROS responsiveness of hydrogel. (**L**) Swelling ratio of hydrogel. (**M**) Degradation of hydrogel in vitro. (**N**,**O**) Kinetics of DOX and SIS3 release from hydrogel under different conditions.

After lyophilizing the obtained hydrogels, samples were prepared and observed via an SEM (Figure 6A). The hydrogels exhibited a characteristic interconnected network structure, which can serve as a biomaterial scaffold to facilitate drug migration–release and nutrient transport, thereby providing a favorable buffering environment for promoting controlled drug release. The gel structure without NPs showed a loose–dense multilayered network resembling muscle tissue. The gel network became more homogeneous with the NP addition, and the NPs were uniformly loaded into the hydrogel network with high dispersion density, facilitating drug loading and sequential release. Confocal laser scanning microscopy confirmed the homogeneous distribution of red fluorescently labeled DOX-U14 and green fluorescently labeled SIS3-CAF within the hydrogel (Figure 6B).

The viscosity-versus-shear rate curves for the hydrogels with and without loaded NPs indicate the significant shear-thinning behavior of the hydrogels irrespective of NP encapsulation (Figure 6C), which facilitates their processing and application. For example, they can be applied for injection/extrusion, as the viscosity is reduced when passing through a needle or nozzle (high shear rate), allowing for effortless and smooth flow. They can also be used for coating because they are easy to spread during brushing or spraying (high shear rate).

The rheological strain sweep results indicate that the NP incorporation significantly enhanced the mechanical strength of the hydrogel network (Figure 6D), implying that the injection-molded Gel-NP scaffold can better resist compression from surrounding tissues, maintain its predetermined shape, and provide improved mechanical support. Specifically, within the linear viscoelastic region, the storage modulus (G′ ≈ 6260 Pa) of the Gel-NPs is considerably higher than that of the neat Gel (G′ ≈ 2140 Pa), indicating that the NPs acted as effective physical crosslinking points or perhaps interacted with the network, greatly increasing the gel rigidity. However, the crossover point of the G′ and G″ (i.e., the gel point) shifts markedly from 105% for the Gel to 48% for the Gel-NPs, suggesting that while NPs enhanced the strength, they also introduced more stress concentration points that may have restricted the dynamic bond (imine and boronate ester bonds) rearrangement capability, causing the network to disintegrate under relatively smaller strains and thereby reducing its resistance to large deformation (toughness).

The frequency sweep results show that both hydrogels exhibited typical solid-like elastic behavior (G′ > G″) over the entire tested frequency range (0.1–100 rad/s), confirming their stable crosslinked network structures (Figure 6E). The NP incorporation significantly enhanced the mechanical properties of the gels. The storage modulus (G′) of the Gel-NPs is much higher than that of the neat Gel across the whole frequency range, consistent with the increased modulus observed in the linear region of the strain sweep and indicating that NPs act as additional physical crosslinking points, effectively strengthening the network and making it more resistant to deformation across various time scales. Notably, the G′ and G″ curves of both gels show relatively flat frequency dependences, especially the G′ curve, suggesting that the network has good stability and limited relaxation mechanisms. The presence of dynamic covalent bonds (imine and boronate ester bonds) provides sufficient crosslinking density, allowing the network to maintain its integrity without flowing or disintegrating even under rapid mechanical perturbations (high-frequency region). This is crucial for retaining the shape and mechanical performance of injectable hydrogels in vivo.

The recovery of the G′ and G″ after subjecting the Gel and Gel-NPs to a large strain that disrupted the hydrogel (130% for Gel; 50% for Gel-NPs) is shown in Figure 6F,G. Although the critical failure strains of the Gel and Gel-NPs are different (130% and 50%, respectively), both gels recovered well in terms of the G′ and G″ when a small strain was applied after high-intensity disruption. This strongly demonstrates that the core driving force of their crosslinked network—the imine and boronate ester bonds—possesses excellent dynamic reversibility and self-healing. The neat Gel network is more flexible and strain-tolerant (higher failure strain point). Therefore, under a large strain of 130%, although the network was substantially stretched and disrupted (bond breakage), once the strain was reduced to a small strain of 2%, the abundant dynamic covalent bonds quickly reassociated, reforming effective crosslinking points and restoring the macroscopic modulus. The NP incorporation significantly increased the rigidity of the Gel-NP network (higher G′) but also introduced more stress concentration points, causing the overall structure to collapse and fail at a smaller strain (50%). Nevertheless, their recovery ability indicates that the NPs primarily serve as inert reinforcing fillers tightly encapsulated by the dynamic covalent bond network, without hindering the dynamic dissociation and reassembly of the imine and boronate ester bonds. Upon stress removal, these dynamic bonds can rapidly recombine, rebuilding a robust network reinforced by the NPs. This recoverable property is essential for injectable applications, as it mimics the behavior of materials that experience high shear and large strain disruption when passing through a narrow needle, followed by a low-stress static environment after injection into the body, indicating that both gels, especially Gel-NPs, can effectively “self-heal” after injection, restoring their mechanical integrity to fulfill their functions as supports or carriers.

Next, we macroscopically verified a series of corresponding properties of the Gel-NPs. The formation–dissociation cycle of dual dynamic covalent bonds within this drug-loaded hydrogel accounts for its self-healing and injectability properties, which are important characteristics. Its excellent injectable and moldable capability allows the hydrogel to be injected into a specific tumor site for in situ gelation, while the self-healing property enables it to withstand external mechanical forces and prolongs its service life. To evaluate the injectability of the hydrogel, precursor solutions A and B were prepared in advance and then injected uniformly and continuously in equal volumes through a double-barrel syringe and a 25 G needle (0.6 mm × 25 mm, TWLB point style) (Figure 6H) to form the letters “U” and “S” (Figure 6I). The prepared hydrogel was cut into two pieces and adhered to the surface of a finger to assess its self-healing behavior. After recontacting the two cut pieces, the hydrogel self-healed and resisted the finger’s bending force without cracking (Figure 6J).

Swelling is an inherent property of hydrogels that affects the release rate of loaded drugs. The Gel-NPs exhibited a relatively higher water absorption rate compared with the neat hydrogel (Figure 6L). The swelling ratio reached equilibrium within 3 h, and this relatively low swelling ratio contributed to maintaining structural stability. Furthermore, the in vitro degradation behavior of the as-prepared hydrogels was investigated in a simulated physiological microenvironment using PBS (pH 7.4) at 37 °C. All hydrogels exhibited continuous degradation behavior after 7 days of incubation (Figure 6M), indicating that they possess excellent degradability, making them suitable for further in vivo applications.

The hydrogel exhibits responsive behavior under different pH and redox conditions. Thus, in this study, the dual-pH/ROS-responsive behavior of the hydrogel was investigated under various conditions (Figure 6K). The hydrogel gradually disintegrated within 7 days of the HCl solution addition (pH 5.5), and a similar responsive behavior was observed after the addition of H_2_O_2_ (1 mM). These results indicate that the hydrogel network is disrupted because of the hydrolysis of dynamic covalent bonds (imine and boronate ester bonds) under acidic and oxidative conditions. Once the network collapses, DOX-U14 and SIS3-CAFs are gradually released. We further analyzed the drug release kinetics of the hydrogel under different conditions (Figure 6N,O). After 24 h of incubation, 20.50% of DOX and 15.86% of SIS3 were released from the hydrogel under acidic conditions (pH 5.5). The release of both drugs was enhanced in the presence of H_2_O_2_, owing to the high sensitivity of the boronate ester bonds to reactive oxygen species (ROS). These results collectively indicate that the hydrogel exhibits a dual responsiveness to acidic pH and elevated ROS levels.

### 3.6. In Vitro Biological Effects of Stimuli-Responsive Hydrogels

**Figure 7:**
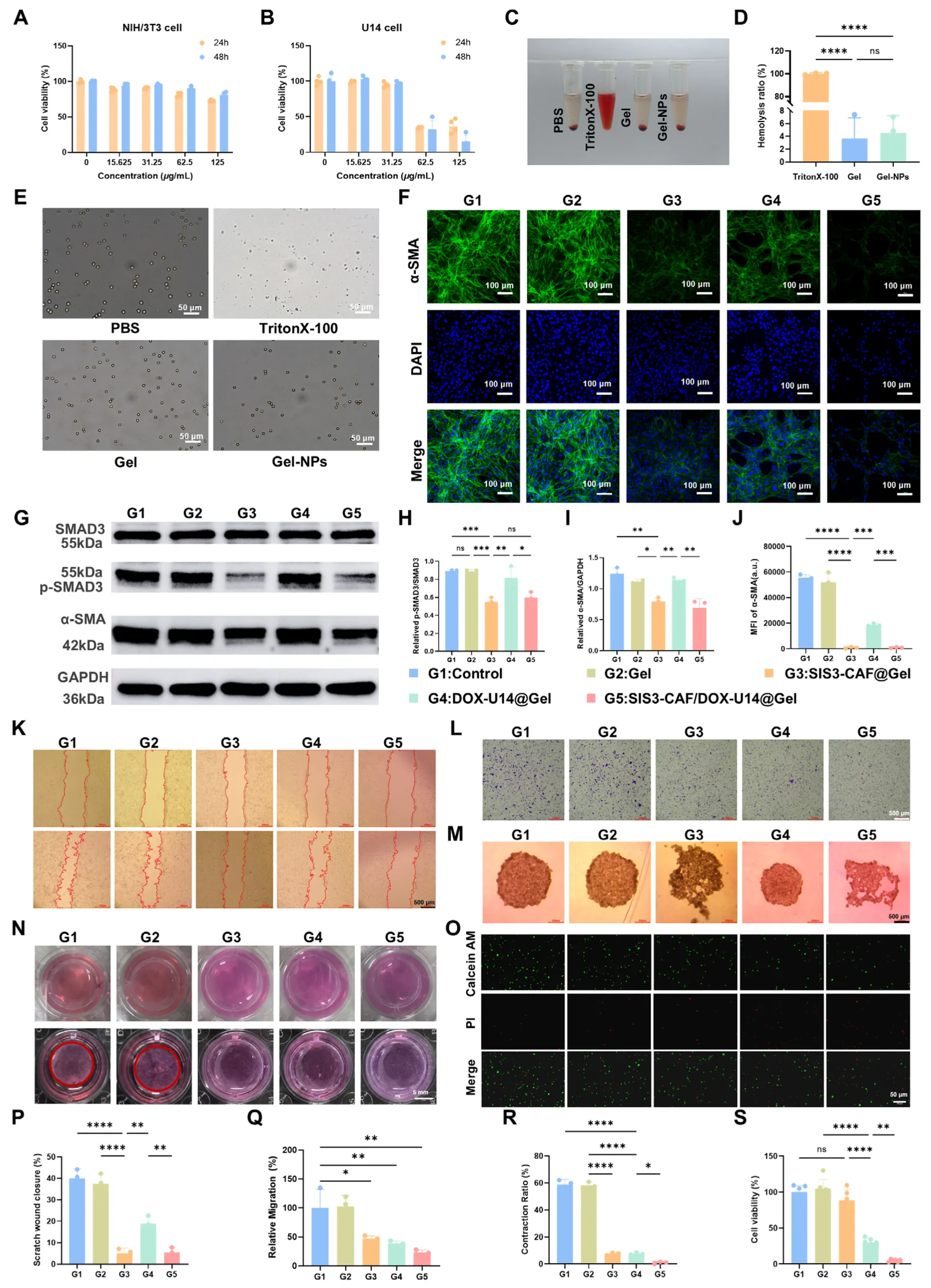
In vitro biological effects of stimuli-responsive hydrogels. (**A**,**B**) Cell viability of NIH/3T3 and U14 cells after incubation with hydrogels for 24 or 48 h, as determined via CCK-8 assay. (**C**–**E**) Representative photographs and statistical analysis of hemolysis assays for hydrogels with and without NPs. Scale bars: 50 μm. (**F**,**J**) Representative images and statistical analysis of α-SMA (green fluorescence) in each treatment group (G1: Control; G2: Gel; G3: SIS3-CAF@Gel; G4: DOX-U14@Gel; G5: SIS3-CAF/DOX-U14@Gel). Scale bars: 100 μm. (**G**–**I**) Western blot images and statistical analysis of SMAD3, p-SMAD3, and α-SMA in CAFs. (**K**,**P**) Representative images and statistical analysis of relative wound-healing degree in wound-healing assays performed on CAFs from each treatment group. Scale bars: 500 μm. (**L**,**Q**) Representative images and statistical analysis of relative migration rate in Transwell migration assays performed on CAFs from each treatment group. Scale bars: 500 μm. (**M**) Representative photographs of fibrotic tumor spheres from each treatment group. Scale bars: 500 μm. (**N**,**R**) Representative images and statistical analysis of relative contraction degree in collagen gel contraction assays performed on CAFs from each treatment group. Scale bars: 5 mm. (**O**) Representative fluorescence images showing live (green)/dead (red) U14 cells after various treatments, stained with calcein-AM/PI. Scale bars: 50 μm (**S**) U14 cell viability for 24 h, as determined via CCK-8 assay in each treatment group. Data are shown as mean ± SD (*n* = 3). Statistical significance was determined by One-way ANOVA with Tukey’s post hoc test. “ns” indicates not significant, * *p* < 0.05; ** *p* < 0.01; *** *p* < 0.001; **** *p* < 0.0001.

After confirming the stimuli-responsive properties of the hydrogels, we evaluated their cytotoxicity in vitro. The neat hydrogel was directly co-cultured with U14 cells and NIH/3T3 cells, and CCK-8 assays were performed after 24 and 48 h (Figure 7A,B). The hydrogel had no significant effect on the NIH/3T3 cell viability at concentrations up to 125 μg/mL. However, the hydrogel induced the death of U14 cells at a concentration of 62.5 μg/m. This differential effect between normal and tumor cells may be partially attributed to the unique TME generated by tumor cell metabolism.

The hemolysis level is one of the important indicators for evaluating the compatibility of biomaterials in vitro [39]. After incubating the Gel and Gel-NPs in PBS containing 5% red blood cells for 1 h, microscopic observation showed that most of the red blood cells remained biconcave and disk-shaped, with an essentially normal morphology and size (Figure 7E). After centrifugation, a large number of intact red blood cells formed a visible precipitate at the bottom of the tubes in the negative control group as well as in the Gel and Gel-NP sample groups, and the supernatants were clear (Figure 7C). In contrast, in the positive control group, extensive red blood cell lysis occurred, the solution turned red, and no obvious precipitate was observed at the tube bottom. The supernatants were measured using a microplate reader for OD values, and the calculated hemolysis rates were (3.65 ± 2.64)% for the neat Gel and (4.52 ± 2.19)% for the Gel-NPs (Figure 7D).

We confirmed that SMAD3 phosphorylation is elevated in CAFs. Next, we evaluated the effect of the SIS3-loaded hydrogel on TGF β/SMAD3 pathway inhibition. The fluorescence distribution of α-SMA was observed under confocal laser scanning microscopy, and the results showed that the green fluorescence of α-SMA was significantly reduced in the experimental groups containing the inhibitor SIS3 (G3: SIS3-CAF@Gel and G5: SIS3-CAF/DOX-U14@Gel) (Figure 7F,J). Meanwhile, the Western blot and quantification results also confirmed the successful inhibition of the TGF β/SMAD3 pathway (Figure 7G–I). Furthermore, the CAF behavior was examined via wound-healing and Transwell assays (Figure 7K,L,P,Q). The enhanced migration and invasion capabilities of CAFs were effectively suppressed in the experimental groups containing SIS3 (G3 and G5), indicating that SIS3 effectively suppresses these malignant behaviors of CAFs without impairing their viability.

Next, we evaluated whether the SIS3-loaded hydrogel disrupted CAF-induced ECM densification, an important feature of the tumor stroma. U14 cells and CAFs were mixed at a 2:1 ratio and co-cultured in U-bottom 96-well plates. After 7 days of co-culturing, the experimental groups without SIS3 (G1: Control; G2: Gel; G4: DOX-U14@Gel) formed highly compact spheroids consisting of tumor cells and fibroblasts (Figure 7M). The spheroids were uniformly dense, round, and well-defined, with few free single cells or small cell clusters around the spheroids. In the DOX-loaded hydrogel group (G4), the diameter and volume of the mixed spheroids were smaller than those in the PBS and neat hydrogel groups (G1 and G2), which is attributed to the cytotoxic effect of DOX; however, ECM densification caused by CAFs reduced the penetration of the chemotherapeutic drug DOX into the tumor spheroids. In contrast, in the SIS3-loaded hydrogel groups (G3 and G5), co-cultured tumor cells and fibroblasts formed loose, grape-like aggregates with obvious intercellular spaces. Moreover, in the group loaded with both SIS3 and DOX, the aggregate volume was significantly reduced, and the cell number was markedly decreased. These morphological differences indicate that SIS3 remodels CAFs, reducing ECM density and further promoting DOX penetration into the tumor spheroids, thereby enhancing the killing efficiency of tumor cells.

We used rat tail tendon collagen type I to simulate the TME in vitro to verify the ability of the drug-loaded hydrogel to remodel the ECM. U14 cells and CAFs were embedded in collagen type I gel at a ratio of 2:1. After 48 h, we observed that active CAFs embedded in the collagen gel significantly induced collagen contraction (G1 and G2), with a marked reduction in the collagen gel area, which is associated with the CAF-mediated adhesion and contraction of the ECM (Figure 7N,R). In contrast, CAF-induced gel contraction was attenuated in the hydrogels loaded with SIS3 and DOX, and the collagen gel area remained almost unchanged, indicating that SIS3 effectively inhibited the contractile capacity of CAFs and reduced mechanical stress within the ECM. Meanwhile, we evaluated the antitumor effect of the drug-loaded hydrogels. The U14 cell viability was assessed using calcein-AM/PI staining and CCK-8 assays (Figure 7O,S). The results showed that SIS3 alone did not directly induce apoptosis or impair cell viability, whereas DOX effectively killed tumor cells, and its effect was synergistically enhanced in the presence of SIS3. These results indicate that SIS3 loaded in the hydrogel effectively reprogrammed CAFs by blocking the TGF-β/SMAD3 signaling pathway, remodeled the dense ECM in the tumor tissue, and thereby enabled better penetration of the loaded chemotherapeutic drug DOX into the tumor interior, enhancing the therapeutic efficacy.

### 3.7. In Vivo Biological Effects of Stimuli-Responsive Hydrogels

**Figure 8:**
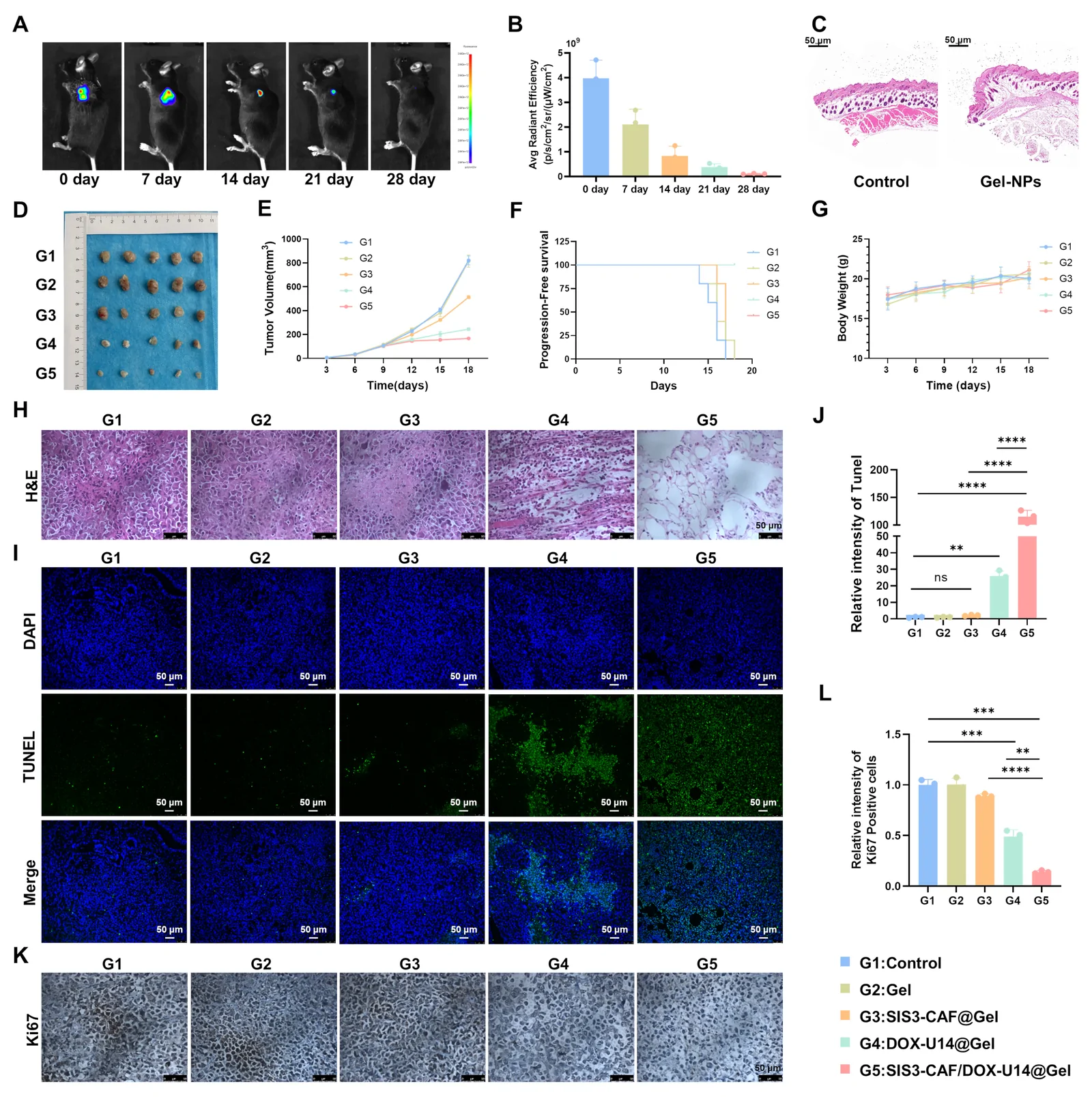
In vivo antitumor effects of stimuli-responsive hydrogels. (**A**) In vivo degradation fluorescence imaging of hydrogel in mouse (0, 7, 14, 21, 28 days). (**B**) Quantitative analysis of fluorescence in degradation images of hydrogel (*n* = 3). (**C**) Images of H&E-stained skin tissue after hydrogel injection (*n* = 3). Scale bars: 500 μm. (**D**) Photographic record of subcutaneous tumor resection in mice (*n* = 5). (**E**) Growth curves of fibrotic tumors in mice (*n* = 5). Scale bars: 50 μm. (**F**) Survival curves of mice in each group after treatment (*n* = 5). (**G**) Body weight change curves of mice in each group after treatment (*n* = 5). (**H**) Representative H&E staining images of tumors from each treatment group (*n* = 3). (**I**,**J**) Representative TUNEL staining images of tumors from each treatment group and statistical analysis of relative fluorescence intensity (*n* = 3). Scale bars: 50 μm. (**K**,**L**) Representative IHC staining images of Ki67 in tumors from each treatment group and statistical analysis of relative content of positive cells (*n* = 3). Scale bars: 50 μm. G1: Control; G2: Gel; G3: SIS3-CAF@Gel; G4: DOX-U14@Gel; G5: SIS3-CAF/DOX-U14@Gel. Data are shown as mean ± SD. Statistical significance was determined by One-way ANOVA with Tukey’s post hoc test. “ns” indicates not significant, ** *p* < 0.01; *** *p* < 0.001; **** *p* < 0.0001.

The excellent performance of the stimuli-responsive hydrogels in vitro prompted us to further explore their in vivo biological effects. Thus, we systematically investigated the biodegradability and biocompatibility of these drug-loaded hydrogels in vivo to evaluate their suitability as local drug delivery platforms. Subcutaneous implantation experiments showed that the hydrogels gradually degraded over 28 days, indicating controllable biodegradability (Figure 8A,B). Specifically, 100 µL of indocyanine green-loaded hydrogel was injected subcutaneously into mice, and the fluorescence signals were monitored periodically using an in vivo imaging system over 28 days. The residual fluorescence intensities were 52.99 ± 12.73% at day 7, 20.84 ± 8.25% at day 14, 9.72 ± 2.72% at day 21, and 2.86 ± 0.35% at day 28, demonstrating the gradual degradation of the hydrogels over time (Figure S3). Furthermore, H&E staining of the skin tissue surrounding the implanted hydrogel showed no signs of necrosis or tissue damage (Figure 8C), further confirming the good local biocompatibility of the hydrogels.

Next, we constructed a subcutaneous xenograft mouse model of fibrotic cervical cancer by mixing U14 cells and CAFs at a ratio of 2:1 to further evaluate the antitumor efficacy in vivo. Tumor-bearing mice were randomly divided into five groups, as follows: G1—control group (PBS injection); G2—Gel group (blank hydrogel injection); G3—SIS3-CAF@Gel group (SIS3-CAF-loaded hydrogel injection); G4—DOX-U14@Gel group (DOX- U14-loaded hydrogel injection); and G5—SIS3-CAF/DOX-U14@Gel group (SIS3- CAF/DOX-U14-loaded hydrogel injection). Tumor growth was monitored after the respective treatments, and no significant difference in tumor growth was observed between the G1 and G2 groups. Thus, the mice were euthanized on day 18, when the maximum tumor volume reached approximately 1000 mm3. We found that the SIS3-CAF/DOX-U14@Gel group exhibited the most significant tumor growth inhibition, followed by the DOX-U14@Gel group, while the SIS3-CAF@Gel group showed the weakest inhibition (Figure 8D–F). Notably, to evaluate the therapeutic efficacy of the drug, we adopted progression-free survival (PFS) as the primary endpoint. Because of the limited observation window and the fact that most mice had not yet reached the progression endpoint by the end of the experiment, the PFS curves showed no statistically significant difference between groups, indicating that SIS3 loaded in the hydrogel synergistically enhances the antitumor effect of DOX. Furthermore, body weight measurements showed no significant differences between the treatment and control groups, and mice in all groups exhibited gradual and consistent body weight increases over time (Figure 8G). H&E and TUNEL staining was used to assess tumor cell death to further confirm the antitumor effect of the drug-loaded hydrogels. The results showed extensive tumor cell death in the SIS3- CAF/DOX-U14@Gel group (Figure 8H–J). In addition, Ki67 IHC staining of tumor tissues confirmed the inhibitory effect of the drug-loaded hydrogel on tumor proliferation (Figure 8K,L). Collectively, these results reveal the advantages and potential of this drug-loaded hydrogel in the treatment of cervical cancer.

**Figure 9:**
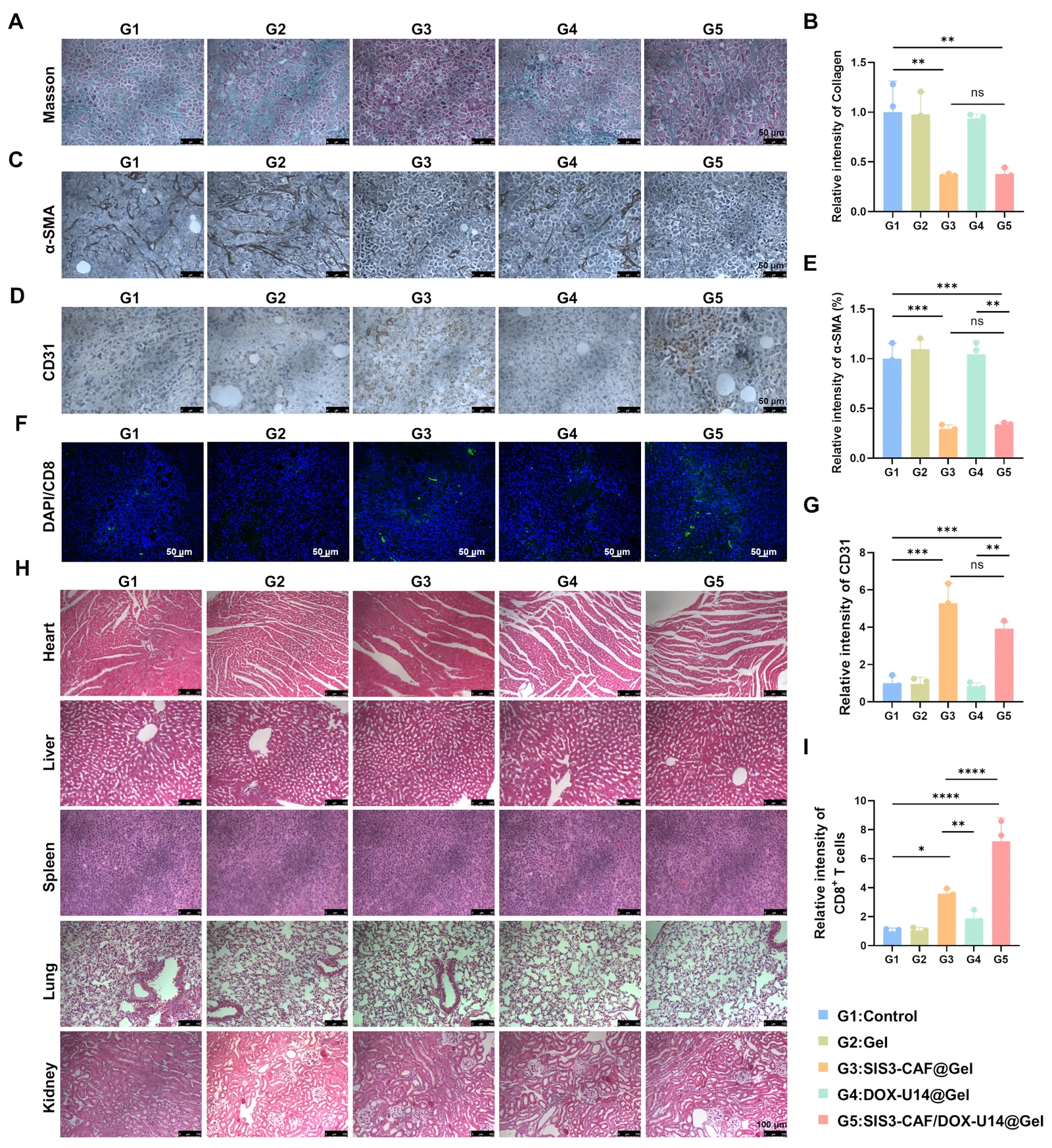
In vivo ECM remodeling effects of stimuli-responsive hydrogels. (**A**,**B**) Representative Masson staining images of tumors from each group after treatment and statistical analysis of relative collagen expression (*n* = 3). Scale bars: 50 μm. (**C**,**E**) Representative IHC staining images of α-SMA in tumors from each group after treatment and statistical analysis of relative α-SMA expression (*n *= 3). Scale bars: 50 μm. (**D**,**G**) Representative IHC staining images of CD31 in tumors from each group after treatment and statistical analysis of relative CD31 expression (*n* = 3). Scale bars: 50 μm. (**F**,**I**) Representative IF staining images of CD8 in tumors from each group after treatment and statistical analysis of relative fluorescence intensity of CD8 (*n* = 3). Scale bars: 50 μm. (**H**) Representative H&E staining images of heart, liver, spleen, lung, and kidney from mice in each group post-treatment (*n* = 3). Scale bars: 100 μm. G1: Control; G2: Gel; G3: SIS3-CAF@Gel; G4: DOX-U14@Gel; G5: SIS3-CAF/DOX-U14@Gel. Data are shown as mean ± SD (*n* = 3). Statistical significance was determined by One-way ANOVA with Tukey’s post hoc test. “ns” indicates not significant, * *p* < 0.05; ** *p* < 0.01; *** *p* < 0.001; **** *p* < 0.0001.

We performed Masson and IHC staining on tumor sections to explore whether the hydrogel loaded with SIS3 enhanced the antitumor effect by reprogramming CAFs. The results showed that collagen deposition was markedly decreased and α-SMA expression was significantly reduced in the SIS3-treated groups (G3 and G5) (Figure 9A–C,E). The dense ECM within fibrotic tumors generates substantial tumor interstitial pressure, leading to abnormal and disorganized blood vessels, which impair blood flow and cause insufficient oxygen supply [40,41]. In existing clinical therapeutic studies, strategies for tumor vascular normalization aim to promote angiogenesis, convert the tumor vasculature to a more normal phenotype, reduce ECM mechanical stress, and improve perfusion efficiency, ultimately improving overall survival [42,43]. Therefore, we examined IHC sections stained for the vascular endothelial marker CD31, and the results revealed that treatment with the SIS3-loaded hydrogel significantly increased the CD31 expression and led to a broader distribution (Figure 9D,G), suggesting that after the SIS3 intervention, the ECM became more organized, successfully alleviating intratumoral matrix pressure. These findings indicate that SIS3 effectively reprograms CAFs, alleviates the dense ECM associated with cervical cancer, and promotes improved therapeutic outcomes. Previous studies have shown that ECM deposition in tumor tissue forms a barrier that blocks CD8^+^ T-cell infiltration and activation, and that blocking TGF-β can reprogram the stromal fibroblasts surrounding the tumor, increase the number of CD8^+^ T cells in the tumor bed, and thereby enhance antitumor immunity [44]. Therefore, we examined the infiltration of antitumor immune cells. Tumor sections from the SIS3-CAF/DOX-U14@Gel group showed the most significant CD8^+^ T-lymphocyte infiltration (Figure 9F,I), indicating that SIS3-induced TME reprogramming also plays a crucial role in promoting immune cell infiltration into the tumor site, an indirect effect that could offer a promising strategy for improving tumor therapeutic outcomes.

Finally, major organs (heart, liver, spleen, lungs, and kidneys) of the mice were collected and subjected to H&E staining, and no obvious pathological changes were observed (Figure 9H). Under the current experimental conditions, these results indicate that the hydrogel and hydrogel-based local drug delivery system did not cause any obvious histological damage.

## 4. Discussion

The treatment of cervical cancer is primarily based on surgical resection, radiotherapy, and chemotherapy [45], with the extent of the disease and the staging system provided by the International Federation of Gynecology and Obstetrics (FIGO; stages I-IV) determining the type of treatment modality [46]. Surgical resection is recommended for patients with early-stage cervical cancer. However, the treatment of advanced cervical cancer is challenging and requires combination therapy, such as radiotherapy and chemotherapy. Platinum-based agents and taxanes are commonly used chemotherapeutics for cervical cancer [47], and chemotherapy alone or in combination with other therapies can improve the overall survival rate of cervical cancer patients [48]. In addition, immunotherapy has also been introduced to eliminate cervical tumors [49]. Nevertheless, cervical cancer cells have developed resistance to radiotherapy, chemotherapy, and immunotherapy [50,51]. The mechanisms of drug resistance in cervical cancer are diverse, such as frequent drug use and molecular pathway dysregulation. Therefore, various novel therapeutic approaches have been adopted to modulate cervical cancer progression, including combination therapy and the application of phytochemicals. However, these strategies have not significantly improved patient prognosis or survival rates. The use and efficacy of these strategies are limited by the toxicity of chemotherapeutic drugs and the difficulty of drug and immune cell infiltration within the fibrotic TME. Consequently, there is a need to introduce new treatment modalities, and our proposed stimuli-responsive hydrogel drug delivery platform is one such approach.

Hydrogels, as novel carriers, hold broad prospects for innovative applications in cervical cancer therapy [52,53], as the porous hydrogel network provides effective support for sustained drug release [38]. The low-pH and high-ROS characteristics of the TME prompted us to design a dual-pH/ROS-responsive hydrogel based on dual dynamic bonds, which can be crosslinked in situ via co-injection of precursor solutions A and B. The selection of PVA, SA, and CSQAS is based on their synergistic functions. PVA provides robust mechanical strength, imparting fundamental toughness and structural stability to the hydrogel, which enables its prolonged retention at the injection site as a local drug depot [54]. SA can be oxidized to OSA, and the aldehyde groups introduced via OSA endow the hydrogel with a dynamic covalent crosslinking capability and environmental responsiveness [55]. CSQAS offers abundant amino groups that can form dynamic imine bonds with the aldehyde groups of OSA. Additionally, 3-APBA can be grafted onto CSQAS via boronate ester bonds, yielding the target polymer CSQAS-APBA. Moreover, the vicinal diol groups of PVA and OSA undergo an ester exchange reaction with the grafted phenylboronic acid groups of CSQAS-APBA to form boronate ester bond crosslinking. In contrast, other commonly used polymers present notable limitations. For example, polyethylene glycol (PEG) lacks aldehyde groups capable of efficiently reacting with amino groups, making it difficult to construct dynamic imine bond networks. Gelatin, as an animal-derived protein, suffers from batch-to-batch variability, pathogen transmission risks, and rapid enzymatic degradation [56]. Hyaluronic acid exhibits poor mechanical strength and does not possess aldehyde crosslinking sites without chemical modification; moreover, its oxidative modification is costly and yields low efficiency [57]. Therefore, the multiple components within the same system form a gel through synergistic crosslinking involving both imine bonds (formed between the aldehyde groups of OSA and the amino groups of CSQAS) and boronate ester bonds (formed between the vicinal diols of PVA/OSA and the phenylboronic acid groups of CSQAS-APBA). The interweaving of these two types of dynamic covalent bonds constructs a three-dimensional network, ultimately achieving the sol–gel transition. Concurrently, this system integrates injectability, self-healing ability, low-pH- and high-ROS-responsive drug release, and biocompatibility without requiring additional crosslinkers or functional additives, thereby rendering it more advantageous for the intended applications. Moreover, this approach facilitates drug storage and transportation before gelation [58], and the presence of dynamic covalent bonds (imine and boronate ester bonds) provides sufficient crosslinking density, enabling the injectable hydrogel to maintain a good shape and mechanical performance in vivo. The dynamic covalent bonds hydrolyze under acidic and oxidative conditions in tumor tissue, leading to the disruption of the hydrogel network and the gradual release of the drug-loaded NPs. Notably, the poor water solubility of SIS3 limits the clinical application of this antifibrotic agent. To overcome this, we cleverly loaded SIS3 into MSNs and coated them with CAF cell membranes, utilizing homologous targeting and cellular endocytosis to precisely deliver SIS3 to CAFs. For targeted delivery to tumor cells, we similarly “camouflaged” DOX by coating it with the same cancer cell membranes, promoting the endocytosis of the chemotherapeutic drug by tumor cells.

In summary, we have made active explorations to overcome the current challenges of drug resistance and chemoresistance in cervical cancer treatment. SIS3-mediated CAF reprogramming actively remodels the ECM to reduce physical and immunosuppressive barriers, promoting deeper drug penetration and improved cytotoxic lymphocyte infiltration. Moreover, embedding DOX in the hydrogel provides a local delivery platform, achieving a high local concentration at the tumor site within the therapeutic window while minimizing systemic exposure. To the best of our knowledge, this is the first study to integrate PVA/SA/CSQAS injectable hydrogels with dual-drug-loaded, cancer cell membrane-coated MSNs for combined chemo- and antifibrotic therapy in cervical cancer. Unlike previously reported hydrogel systems that either lack active targeting or rely on single-stimulus-responsive release [59], our platform uniquely combines homologous targeting, dual pH/ROS responsiveness, and stromal modulation within a single locally injectable formulation. This dual-pronged approach differs from existing monotherapies and offers a rational synergistic strategy for the treatment of advanced or recurrent cervical cancer. Moreover, this minimally invasive approach via vaginal injection holds promise for providing a fertility-sparing strategy for cervical cancer patients of childbearing age. However, we acknowledge several limitations in the present study. The use of the murine U14 cell line and subcutaneous syngeneic model, while enabling evaluation in immunocompetent mice, does not fully reflect the genetic heterogeneity of human cervical cancer or the orthotopic tumor microenvironment. Validation in human cervical cancer cell lines and orthotopic or patient-derived xenograft models is needed to strengthen the translational relevance. Additionally, comprehensive pharmacokinetic and biodistribution studies are required to fully characterize the in vivo performance of our platform. Despite these limitations, this work provides a solid proof of concept for the injectable platform in cervical cancer therapy and offers valuable insights for future translational development.

## 5. Conclusions

In conclusion, we constructed a novel dual-pH/ROS-responsive injectable hydrogel drug delivery platform. Based on bioinformatics analysis, we demonstrated that hyperactivated CAFs in cervical cancer are positively correlated with elevated ECM protein expression, ultimately leading to a dense tumor ECM and exacerbating chemoresistance. To address this, we developed a novel dual-dynamic bond-based hydrogel with multiple functions, including dual pH/ROS responsiveness, injectability, and self-healing ability, in which the loaded cell membrane-coated NPs possess a certain targeting capability. Leveraging the unique role of CAFs in tumor progression, we designed and fabricated CAF membrane-coated NPs for targeted SIS3 delivery, which can reprogram CAFs and reduce their activity, thereby downregulating ECM expression and further inhibiting fibrosis. Following CAF reprogramming and ECM remodeling, improved vascular organization was observed, which may indicate a trend toward vascular normalization. Additionally, enhanced immune cell recruitment was noted, which might have contributed to the observed antitumor effects. This CAF targeting strategy avoids the potential consequences of direct CAF depletion, such as cancer invasion and metastasis. At the same time, it facilitates the deep delivery of chemotherapy drugs coated with homotypic cancer cell membranes and further immune cell penetration via TME remodeling. Thus, this dual-pH/ROS-responsive injectable hydrogel-mediated co-deslivery system targeting CAFs and cancer cells provides a promising strategy for the clinical treatment of cervical cancer.

## Data Availability

The original contributions presented in this study are included in the article/Supplementary Material. Further inquiries can be directed to the corresponding authors.

## Supplementary Materials

The following supporting information can be downloaded at: https://www.mdpi.com/article/10.3390/pharmaceutics18091115/s1, Figure S1: Characterization of membrane-coated nanoparticles; Figure S2: Stability evaluation; Figure S3: Quantitative analysis of remaining fluorescence intensity for in vivo hydrogel degradation.

## Abbreviations

The following abbreviations are used in this manuscript:

3-APBA	3-Aminophenylboronic Acid
α-SMA	Alpha-Smooth Muscle Actin
ACTA2	Actin Alpha 2, Smooth Muscle
ANOVA	Analysis of Variance
CAFs	Cancer-Associated Fibroblasts
CCK-8	Cell Counting Kit-8
CD31	Cluster of Differentiation 31
CD8	Cluster of Differentiation 8
CESC	Cervical Squamous Cell Carcinoma
COL	Collagens
COL10A1	Collagen Type X Alpha 1 Chain
COL1A1	Collagen Type I alpha 1 chain
CSQAS	Chitosan Quaternary Ammonium Salt
CTAC	Cetyltrimethylammonium Chloride
Cy3	Cyanine 3
DAPI	4′,6-Diamidino-2-Phenylindole
DEGs	Differentially Expressed Genes
DLS	Dynamic Light Scattering
DMEM	Dulbecco’s Modified Eagle Medium
DMSO	Dimethyl Sulfoxide
DOX	Doxorubicin
DOX-U14	DOX-MSN@cancer Cell Membrane
DPI	Dispersity
ECM	Extracellular Matrix
FAP	Fibroblast Activation Protein
FBS	Fetal Bovine Serum
FIGO	International Federation of Gynecology and Obstetrics
FITC	Fluorescein Isothiocyanate
FN	Fibronectin
FN1	Fibronectin 1
FTIR	Fourier Transform Infrared Spectroscopy
G′	Storage Modulus
G″	Loss Modulus
Gel	Hydrogel
Gel-NPs	Nanoparticle-Loaded Hydrogel
GEO	Gene Expression Omnibus
GEPIA2	Gene Expression Profiling Interactive Analysis 2
GPC	Gel Permeation Chromatography
GTEx	Genotype-Tissue Expression
HCl	Hydrochloric Acid
H&E	Hematoxylin and Eosin
HRP	Horseradish Peroxidase
IHC	Immunohistochemical
IUPAC	International Union of Pure and Applied Chemistry
Ki67	Ki-67 Antigen
MSNs	Mesoporous Silica Nanoparticles
NPs	Nanoparticles
OD	Optical Density
OSA	Oxidized Sodium Alginate
PEG	Polyethylene glycol
PBS	Phosphate-Buffered Saline
PDI	Polydispersity Index
PS	Penicillin-Streptomycin
p-SMAD3	Phosphorylated SMAD3
PVA	Polyvinyl Alcohol
RBCs	Red Blood cells
ROS	Reactive Oxygen Species
SA	Sodium Alginate
SEM	Scanning Electron Microscope
SIS3	Specific Inhibitor of SMAD3
SIS3-CAF	SIS3-MSN@CAF Membrane
Smad	Small Mother Against Decapentaplegic
SMAD3	SMAD Family Member 3
TCGA	The Cancer Genome Atlas
TEA	Triethanolamine
TEM	Transmission Electron Microscope
TEOS	Tetraethyl Orthosilicate
TGF-β	Transforming Growth Factor-β
TME	Tumor Microenvironment
TUNEL	Terminal Deoxynucleotidyl Transferase dUTP Nick End Labeling
UV-Vis	Ultraviolet-Visible
XRD	X-Ray Diffraction
