# Supplementary material for: A Locally Injectable, pH/ROS-Responsive Hydrogel Platform for Combination Therapy of Cervical Cancer with Anti-Fibrotic and Chemotherapeutic Agents
Source: Pharmaceutics. 2026 Sep 4;18(9):1115. doi: 10.3390/pharmaceutics18091115 (PMC13610339; doi:10.3390/pharmaceutics18091115)
Supplement: Supplementary file 1 [file pharmaceutics-18-01115-s001.zip › pharmaceutics-4397202-supplementary.pdf]

Supplementary Materials: A Locally Injectable, pH/ROS-Responsive Hydrogel Platform for Combination Therapy of Cervical Cancer with Anti-Fibrotic and Chemotherapeutic Agents

Qian Chen, Hui Yang, Meili Pei, Yanxia Sun, Yubei Li, Sen Yu and Xiaofeng Yang

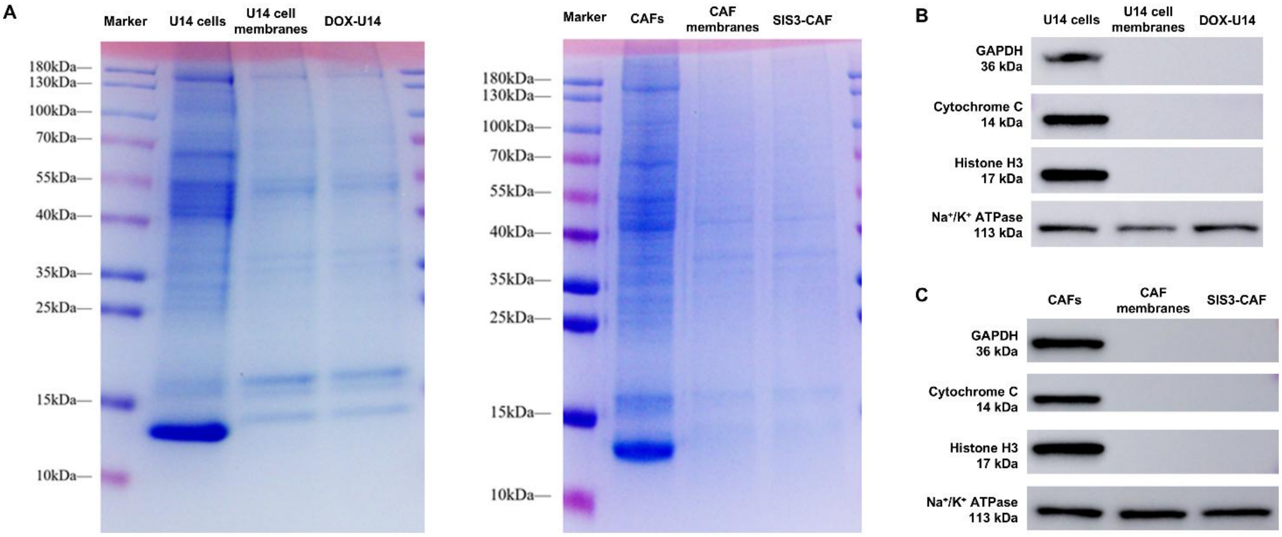

Figure S1. Characterization of membrane-coated nanoparticles. (**A**) SDS-PAGE analysis of cells, cell membranes and nanoparticles. (**B**) Western blot analysis of U14 cells, U14 cell membranes and DOX-U14. (**C**) Western blot analysis of CAFs, CAF membranes and SIS3-CAF.

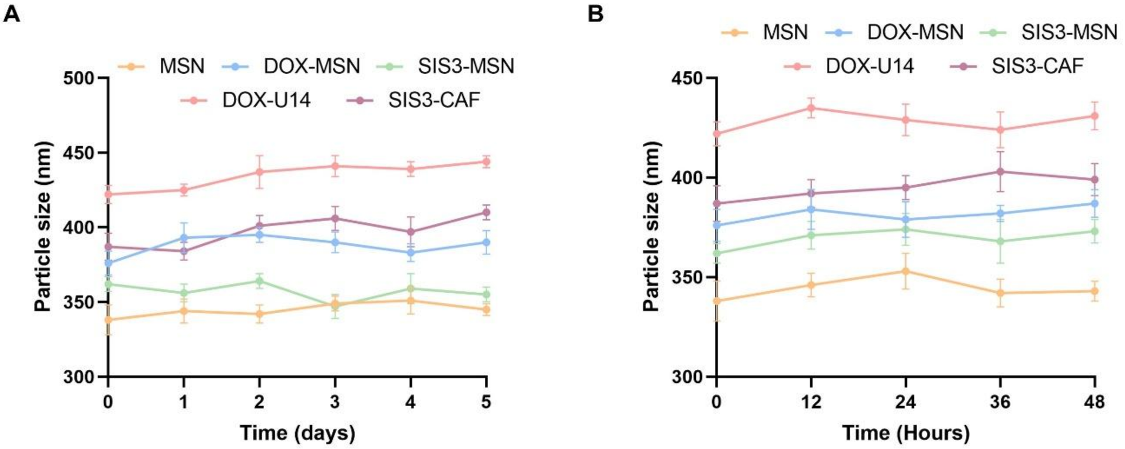

Figure S2. Stability evaluation. (**A**) Changes in nanoparticle size over 5 days in PBS at 4°C (n = 3). (**B**) Changes in nanoparticle size over 48 hours in 20% FBS at 37°C (n = 3).

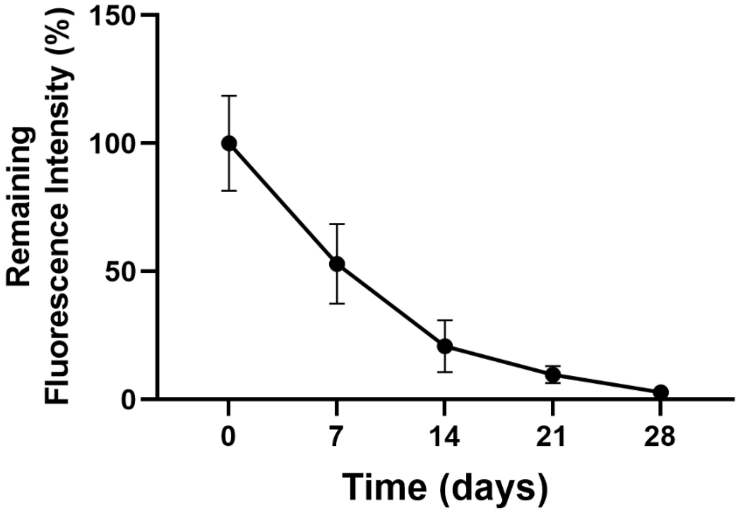

Figure S3. Quantitative analysis of remaining fluorescence intensity for in vivo hydrogel degradation.
